# Copper is an intestinal habitat filter affecting the gut microbiota interactions with *Salmonella* Typhimurium

**DOI:** 10.1186/s40168-025-02322-4

**Published:** 2026-03-28

**Authors:** Rafał Kolenda, Marwa M. Hassan, Ainhoa Arrieta-Gisasola, Abioseh Kamara, Rebecca Ansorge, Katarzyna Sidorczuk, Luke Acton, Gaëtan Thilliez, Dave J. Baker, Michał Burdukiewicz, Mark D. Stares, Hilary P. Browne, Gwenaelle Le Gall, Ricardo C. Torres, Alfredo Chavez-Arroyo, John Garrett, Mark P. Stevens, Trevor D. Lawley, Andreas J. Bäumler, Roberto La Ragione, Falk Hildebrand, Robert A. Kingsley

**Affiliations:** 1 Quadram Institute, Norwich, UK; 2 Department of Biochemistry and Molecular Biology, Faculty of Veterinary Medicine, Wrocław University of Environmental and Life Sciences, Wrocław, Poland; 3 School of Veterinary Medicine, University of Surrey, Guildford, Surrey UK; 4 University of the Basque Country, Vitoria-Gasteiz, Spain; 5 University of East Anglia, Norwich, UK; 6 School of Biotechnology, Dublin City University, Dublin, Ireland; 7 Clinical Research Centre, Medical University of Białystok, Białystok, Poland; 8 Host-Microbiota Interactions Laboratory, Wellcome Sanger Institute, Hinxton, UK; 9 Roslin Institute, Edinburgh, UK; 10 UC Davis, Davis, CA USA; 11 Sparsholt College, Sparsholt, Hampshire SO21 2NF UK; 12 School of Biosciences, University of Surrey, Guildford, Surrey UK; 13 Earlham Institute, Norwich, UK

## Abstract

**Background:**

Foodborne pathogens, including *Salmonella enterica* serovar Typhimurium (*S*. Typhimurium), pose a significant threat to both human health and livestock productivity. The pandemic *S.* Typhimurium ST34 clone acquired a genomic island (SGI-4) conferring high copper resistance, an adaptation relevant in the context of the widespread use of copper sulphate at therapeutic levels in pig farming. We investigated how high dietary copper influences the piglet gut microbiota and *Salmonella*-microbiota interactions that may explain the global spread of *S.* Typhimurium ST34.

**Results:**

An on-farm study combined with faecal shotgun metagenomics revealed that several potential *Salmonella* competitor species, including *Bifidobacterium*, *Escherichia*, and *Lactobacillus*, were less abundant in piglets on high-copper diets. Anaerobic and aerobic culturing alongside whole genome sequencing of 131 species and copper sulphate susceptibility testing identified copper resistance gene acquisition in selected microbes, particularly within *Escherichia*. Niche competition assays demonstrated that copper resistance is critical for inter-species competition under high-copper conditions, with *Salmonella*’s Type VI Secretion System providing a distinct advantage over *Escherichia* in the copper-modified niche.

**Conclusions:**

Our findings suggest that copper supplementation alters the piglet gut environment, impacting competitive dynamics between pathogenic and commensal bacteria, likely to influence the zoonotic transmission of pathogens.

Video Abstract

**Supplementary Information:**

The online version contains supplementary material available at 10.1186/s40168-025-02322-4.

## Introduction

*Salmonella enterica* is a leading zoonotic cause of foodborne illness worldwide, with livestock a primary source of infection. Consequently, an understanding of the impact of livestock husbandry practices on the colonisation of livestock by this pathogen is crucial to devise strategies to reduce the burden of salmonellosis. *Salmonella enterica* serovar Typhimurium (*S*. Typhimurium) is one of the most common causes of human salmonellosis. In 2023, *S*. Typhimurium accounted for 14% of reported human cases in the EU and UK (5.1% monophasic) (European Food Safety, European Centre for Disease, and Control [28]. Similar trends have been observed in the USA, where *S*. Typhimurium caused 15% of domestic outbreaks and 7% of all *Salmonella* infections [90]. *S*. Typhimurium prevalence is also a concern for livestock farming due to its impact on animal welfare and productivity [9]. Over the past 70 years, five different *S*. Typhimurium pandemic clones have dominated human infections, each for approximately 10–15 years [79]. Epidemiological data from the last 15 years indicate that the previously dominant DT104 clone has been replaced by a new pandemic clone, *S*. Typhimurium ST34 [76]. This clone first appeared in the epidemiological record in Europe around 2005 and became dominant in the UK by 2010 [76]. *S*. Typhimurium ST34 is characterised by resistance to ampicillin, streptomycin, sulfamethoxazole, and tetracycline [6] and acquisition of *Salmonella* genomic island 4 (SGI-4), conferring resistance to copper salts [10]. Furthermore, the acquisition of prophages encoding a virulence factor SopE contributed to the clonal expansion of *S*. Typhimurium ST34 [95].

Between 2015 and 2019, pork was the primary source of *S*. Typhimurium outbreaks in the EU [19]. A meta-analysis of *Salmonella* serovars in animal-based foods identified Typhimurium, and less frequently Derby, as the most prevalent serovars in pork across Europe, Oceania, Asia, and North America [32]. Pork constitutes over 30% of consumed meat in the world (source: UN-FAO) and demand has been steadily increasing over the past century (*World Agriculture: Towards 2015/2030, An FAO Study* [101]. This increased demand has led to intensified livestock production [67]. Growth promoters, including antibiotics, administered with feed became common practice in the latter half of the twentieth century to improve animal growth and fattening [26]. However, the increasing resistance to antibiotics used as growth promoters in zoonotic pathogens, including *Salmonella*, and the associated risk to human health was recognised in the 1960 s [2]. This led to bans on the use of antibiotics as growth promoters in the EU (2006), the USA (2017), and China (2020). Following these restrictions, livestock producers increasingly relied on copper salts as growth promoters [11], which coincided with the emergence of copper-resistant *S*. Typhimurium ST34 as a pandemic clone. Copper is an essential micronutrient required in feed (~10 ppm), but at elevated concentrations routinely used in pig production for 4 weeks after weaning (>150 ppm) it is also a potent antimicrobial ((FEEDAP) [30]. The use of copper has the advantage that it is not used in human medicine, but the emergence of copper-resistant *S*. Typhimurium ST34 raises the question of whether its use is still effective for controlling *Salmonella* in pigs. Furthermore, the observation that pigs on a copper-supplemented diet cleared ST34 infections more slowly [7]and had a greater burden of ST34 2 days post-infection [3] than pigs on a conventional diet suggests that there may also be an increased risk to food safety.

*S*. Typhimurium pathogenicity in livestock and humans is characterised by inflammatory gastroenteritis [81]. The host inflammatory response to *Salmonella* infection disrupts the microbiota and creates a nutrient-rich niche favourable for *Salmonella* growth. Beneficial effects of growth promoters in piglets includes the inhibition of potential pathogens such as *S. enterica* and enterotoxigenic *Escherichia coli*, as well as improved intestinal health, characterised by reduced crypt depth and increased villus height [94, 108]. Disrupting the microbiota through antibiotic pretreatment increases host susceptibility to *Salmonella* infection, highlighting the importance of a healthy microbiota in conferring colonisation resistance [5, 82]. Therapeutic levels of copper affect the piglet microbiota, altering the abundance of *Escherichia/*coliforms, *Bifidobacterium*, *Lactobacillus*, and other microbial groups [11, 27].

We previously reported that *sil*/*pco* clusters encoded on SGI-4 provide an advantage for the survival of the *Salmonella* ST34 pandemic clone in vitro during anaerobiosis in the presence of CuSO_4_ [10]. This indicated that copper resistance might be an important factor for *Salmonella* survival in the anaerobic environment of the gut of weaned piglets and, as a result, its maintenance in the food chain and transmissions to humans. It has been previously demonstrated that *Salmonella* competes with the intestinal microbiota during host colonisation and that the microbial community composition might be altered by copper supplementation of feed. We therefore tested the hypothesis that copper supplementation at therapeutic levels (150 ppm) routinely used in pigs alters the gut microbiota composition in piglets and changes *Salmonella*-microbiota competition dynamics.

## Results

### High-copper diet affects the relative abundance of a minor subset of the microbiota in the gut of weaned piglets

**Fig. 1:**
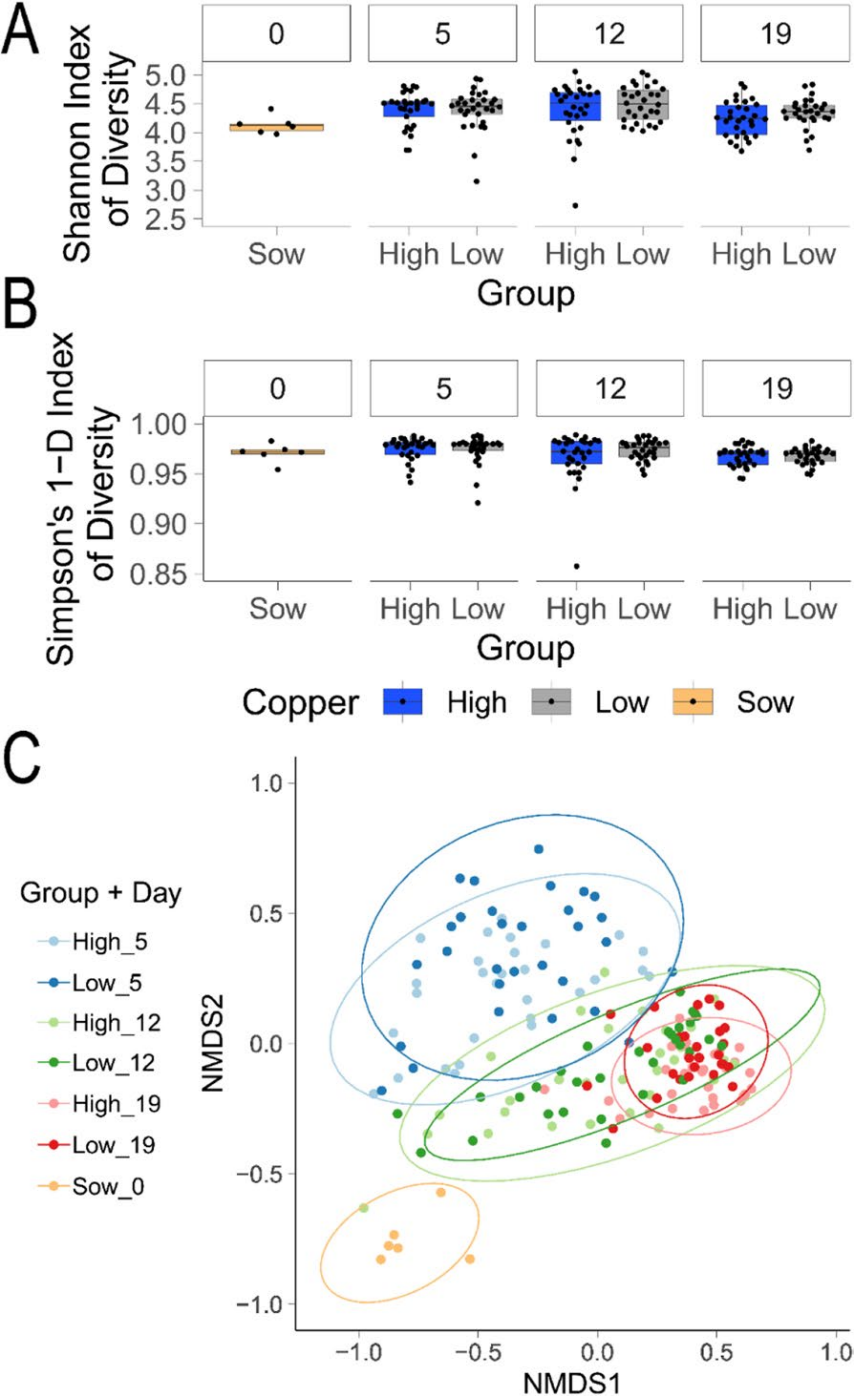
Effect of copper supplementation on pig microbiota communities using shotgun metagenomics. **A** Shannon Index of Diversity and **B** Simpson’s 1-D Index of Diversity. Animal groups are shown on the bottom x-axis and animal study day on the top x-axis. Values for each animal in the group in timepoint are shown as a single point. **C** Non-metric multidimensional scaling (NMDS) using Bray-Curtis dissimilarity showing dissimilarity of species composition between groups during pig farm study. Ellipses for each group display a 95% confidence level for a multivariate *t*-distribution of each group

Previous studies using culture-dependent methods or lower-resolution amplicon sequencing provided valuable insight into the effect of therapeutic copper supplementation (150 ppm) in feed on microbiota composition [48, 107]. We used a metagenomic approach to provide a species-level resolution of changes to the bacterial community. A farm study was designed to replicate the common practice of copper supplementation of feed in the first week of weaning to characterise its effect on the gut microbiota of piglets. During this period, the gut microbiome is at its most turbulent as it adapts to the change in diet from milk to a commercial solid feed. We identified a total of 748 metagenomic species among all samples. Supplementation of feed with 150 ppm copper for 2 weeks did not lead to significant changes in species richness reflected in similar Shannon Index of Diversity and Simpson’s 1-D Index of Diversity for piglets on high (150 ppm) and low (10 ppm) copper diet (Fig.1A, 1B). Assessment of species composition dissimilarity revealed minor differences between therapeutic and nutritional levels of copper at study day 19 (ANOSIM statistic *R* = 0.13, *p* < 0.001). Comparison of dissimilarity of samples prior to changes in copper supplementation on day 5 (high and low) and day 19 (high and low) after 2 weeks on the altered diet revealed moderate differences (ANOSIM statistic *R* = 0.6, *p* < 0.001), indicating the dynamic nature of the microbial communities during the post-weaning period regardless of copper supplementation (Fig. 1C).

**Fig. 2:**
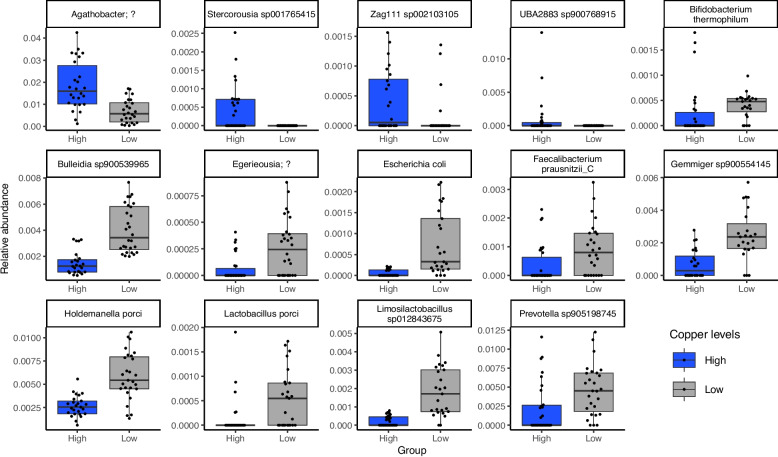
Relative abundance of species with significant differences between high and low copper supplementation at study day 19. Relative abundance of species in all faecal samples for piglets from the farm study. Animal groups are shown on the bottom x-axis and coloured as shown on the legend and bacterial species on the top x-axis. Relative abundance is shown on the y-axis. Each dot represents relative abundance for one piglet from one group at day 19. Outliers were removed. The plot with all datapoints is shown in Fig. S4

The relative abundances of phyla, genera, and species were compared to investigate which microbiota are responsible for minor differences in microbial composition between high and low copper supplementation. At the phylum level, Desulfobacteriota I at study day 12 exhibited decreased abundance on high-copper diet (Wilcoxon test, *p* < 0.05, Figs. S1, S2). This phylum included the genus *Desulfovibrio* (Fig. S3), but this genus alone did not reach statistical significance (Wilcoxon test, *p* = 0.084). Increased abundance of the phylum Cyanobacteria was observed in piglets on high-copper diet at study day 19 (Wilcoxon test, *p* < 0.05) (Figs. S1, S2), reflected in an increased abundance of *Stercorousia sp001765415*, *UBA2883 sp900768915*, and *Zag111 sp002103105* species at day 19 (Wilcoxon test, *p* < 0.05) (Fig. 2, Fig. S4). The relative abundance of phylum Pseudomonadota increased with therapeutic levels of copper in the feed of piglets (Wilcoxon test, *p* < 0.05) (Figs. S1, S2), but investigation of changes within this phylum on the genus and species level revealed that genus *Succinivibrio* increased in relative abundance in piglets on high-copper diet (Wilcoxon test, *p* < 0.05), while genus *Escherichia* and species *Escherichia coli* decreased in abundance (Fig. 2, Figs. S4, S5) (Wilcoxon test, *p* < 0.05). Overall, the high-copper diet affected the relative abundance of 14 species on day 19 (Fig. 2, Fig. S4). Four species belonging to the genus *Agathobacter*, *Stercorousia*, *UBA2883*, and *Zag111* had an increased relative abundance on high-copper diet. Among ten species with decreased abundance on high-copper diet were bacterial species belonging to genus *Bifidobacterium*, *Lactobacillus*, *Gemmiger*, *Holdemanella*, *Faecalibacterium*, and *Prevotella*. Together, our data suggests that copper has only a minor effect on the microbiota composition, but these effects may be biologically significant based on the effect on bacterial taxa with established roles in the healthy microbiome and exclusion of pathogens.

### Cultured pig gut microbiota exhibit variation in copper susceptibility

**Fig. 3:**
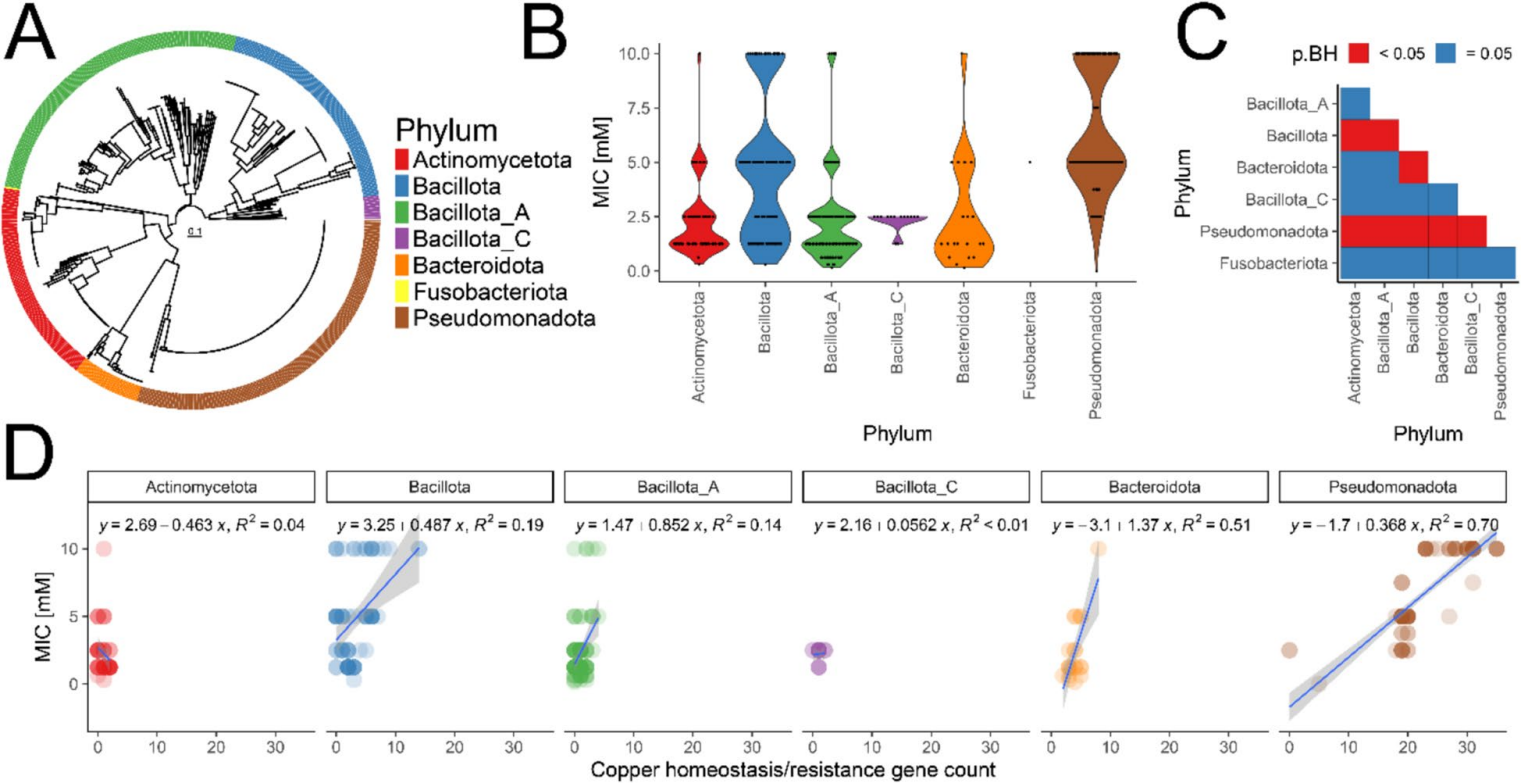
Copper sulphate susceptibility testing of cultured pig microbiota. **A** Phylogenetic tree based on alignment of 120 phylogenetically informative markers concatenated of all cultured bacterial isolates in this study annotated with phylum. **B** Violin-dotplot with copper sulphate MIC in cultured pig bacteria. Microbial phyla are shown on the x-axis and copper sulphate MIC on the y-axis. Violin colours correspond to phyla and are shown on the legend. Each dot represents MIC value for one bacterial isolate. **C** Heatmap with statistical comparisons of copper sulphate MIC differences between phyla. Microbial phyla are shown on the x-axis and y-axis. Statistically significant comparisons are marked with red coloured tile (Wilcoxon test, *p* < 0.05). **D** Correlation of copper homeostasis/resistance gene count with copper sulphate MIC. Dotplot with fitted linear model for correlation of copper homeostasis/resistance gene count with copper sulphate MIC. Gene count is shown on the x-axis and copper sulphate MIC on the y-axis. Linear model was used to fit a line (blue) based on available datapoints and 0.95 confidence interval was plotted (grey). Model fit equation and *R*^2^ values were annotated for each phylum

To directly assess the copper sensitivity of pig gut microbiota and enable identification of copper resistance or homeostasis genes, we cultured bacterial isolates from faecal samples from piglets and sows. Overall, 641 isolates encompassing 131 species belonging to five phyla were cultured to purity and their whole genome sequence was determined (Fig. 3A, Fig. S6, Table S1). Thirty-nine species were not previously described, and taxonomic analysis was performed with Type (Strain) Genome Server (TYGS) (Dataset S1). Of the 131 cultured species, 107 were detected in pig metagenomic reads in this or previous studies [40, 103] (Figs. S7, S8). Ten of 24 species not detected in metagenomic reads were previously found in the animal gastrointestinal tract or faeces (five in pigs) (Table S2). Another ten represent new species or genera not previously isolated. Three species were associated with pigs through Microbeatlas [61] and one remaining species—*Bacillus_A bombysepticus*—belongs to the *B. cereus* group.

Representative strains for each species were selected, totalling 383 strains, and copper sulphate MIC screen was performed on 369 (Dataset S2). Overall, Pseudomonadota, represented mainly by *E. coli*, accounted for the highest proportion of strains with high MIC compared to other phyla (Fig. 3B, C). Additionally, isolates of Bacillota differed in MIC when compared with Actinomycetota, Bacillota_A, and Bacteroidota. Increased resistance to copper sulphate was associated with a higher count of genes associated with copper homeostasis and/or resistance in Pseudomonadota and Bacteroidota (Fig. 3D). By combining high-throughput culturing and whole genome sequencing, we characterised the post-weaning piglet gut microbiota and its resistance to copper sulphate.

### *Escherichia coli* copper resistance clusters are on mobile genetic elements distinct from copper-encoding SGI-4 in *S*. Typhimurium ST34

**Fig. 4:**
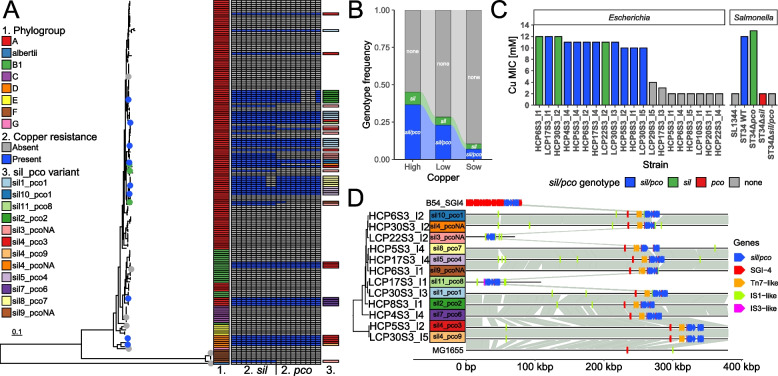
*Escherichia* isolated from pigs and copper resistance. **A** Phylogenetic tree based on core genome SNPs of 174 *Escherichia* isolates in this study annotated with phylogroup, *sil*/*pco* genes, and *sil*/*pco* gene cluster variants. Strains selected for copper sulphate broth microdilution assays are marked with blue (*sil/pco*-positive, *n* = 9), green (*sil*-positive, *n* = 3), or grey dot (*sil/pco*-negative, *n* = 8). **B** Alluvial plot with frequency of copper resistance clusters in *Escherichia* from sows and piglets on high- and low-copper diet. Groups are shown on the x-axis and genotype frequency on the y-axis. Colours correspond to genotype and are labelled with text on the corresponding bars. **C** Barplot with copper sulphate MIC in selected strains of *Escherichia* in anaerobic conditions. Isolate names are shown on the bottom x-axis and genera on the top x-axis. Copper sulphate MIC are shown on the y-axis. Colours correspond to genotype and are shown on the legend. **D** Comparison of *sil*/*pco* clusters with SGI-4 and plasmid/chromosomal localisation of *sil*/*pco* clusters in *Escherichia*. Base position in the sequences is shown on the x-axis (to 380 kbp) and isolate name on the y-axis. Region homologuos between sequences are connected with grey lines. Localisation of SGI-4 genes (not including *sil*/*pco*) is shown with blue squares. Localisation of Tn7-like genes is shown with orange squares. Phylogenetic tree based on core genome SNPs of 12 *Escherichia* isolates annotated with *sil*/*pco* sequence variants

Having observed that a subset of Pseudomonadota exhibited higher copper sulphate MICs and was associated with an increased number of copper homeostasis/resistance genes, we investigated the genetic determinants underlying this phenotype. Nearly all of 174 *Escherichia* isolated during this study encoded well-characterised copper homeostasis genes including *copA*, *cueOR*, *cusABCFRS*, *ndh-2*, and *rclA*. The copper resistance gene clusters *sil/pco* or *sil* alone were identified in 45 and 11 isolates, respectively (Fig. 4A). The frequency of copper resistance genes *sil*/*pco* or *sil* alone was greater in isolates from piglets on a high-copper diet when compared to piglets on a low-copper diet (Fig. 4B, chi-squared test, *p* = 0.05563) or sows (chi-squared test, *p* < 0.005). When the sequence diversity of *sil* and *pco* clusters was investigated, 11 *sil* variants and 9 *pco* variants were identified, resulting in a total of 12 *sil/pco* sequence variants. Copper sulphate MIC determined by the broth microdilution method on *E. coli* isolate subset revealed that all *sil/pco* variants provided similar levels of resistance to SGI-4-encoded *sil/pco* of *S.* Typhimurium ST34 (Fig. 4C). All *Escherichia* isolates without *sil*/*pco* clusters had MIC that was at least 6 mM lower than *Escherichia* with *sil/pco*. Long read sequencing revealed that only two of the *sil*/*pco* sequence variants were plasmid-encoded. All ten chromosome-encoded *sil*/*pco* variants were integrated into one locus and associated with a Tn7-like transposon (Fig. 4D). The *sil* cluster on a plasmid in strain LCP22S3_I2 was surrounded by IS1-like transposable element genes, and the *sil/pco* cluster on a plasmid in strain LCP17S3_I1 was found in proximity to IS3-like genes.

The siderophore yersiniabactin has also been implicated in conferring additional tolerance to copper, albeit in low micromolar concentrations [20]. We therefore investigated the presence of this and the related siderophore system aerobactin, within the whole genome sequence of *E. coli* isolates. A total of 23 isolates encoded genes encoding the yersiniabactin system and 17 isolates encoded the aerobactin system (Fig. S9). None of the strains encoding the *sil* and *pco* genes encoded either siderophore system. One strain encoding yersiniabactin but lacking *sil* and *pco* genes had an MIC similar to other strains lacking all these systems, consistent with previous reports of yersiniabactin having a modest impact on copper resistance relative to that imparted by *sil* and *pco* [20].

### Presence of copper sulphate alters the outcome of interaction between *S.* Typhimurium and *E. coli*

**Fig. 5:**
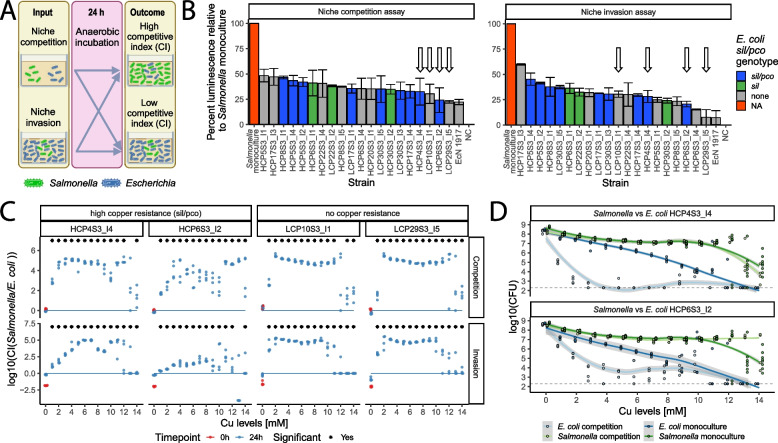
Competition between *Salmonella* and *E. coli* in copper sulphate-altered niche. **A** Schematic representation of niche competition and invasion assays in anaerobic conditions. 1 × 10^6^ CFU of luminescent *Salmonella* and 20 µl or 180 µl of *E. coli* were used for the luminescence competition and invasion assays, respectively. 1 × 10^6^ CFU of *Salmonella* and 1 × 10^6^ CFU or 50 µl of *E. coli* were used for the competition and invasion assays with copper supplementation, respectively. **B** Barplot with the effect of *Escherichia* on *Salmonella* luminescence. Isolate names are shown on the bottom x-axis and assay type on the top x-axis. Luminescence relative to *Salmonella*-only cultures (percent) is shown on the y-axis. Colours correspond to genotype and are shown on the legend. Strains selected for further testing are marked with arrows. **C** Competitive index between *Salmonella* and *E. coli* in the competition and invasion assays with copper supplementation. Copper sulphate concentrations [in mM] are shown on the bottom x-axis and *E. coli* strain name on the top x-axis. Log10 competitive index (CI) between *Salmonella* and *E. coli* CFUs is shown on the left y-axis and the assay type on the right y-axis. The line marks values where CI equals 1. **D** CFUs of *Salmonella* and *E. coli* strain HCP4S3_I4 or HCP6S3_I2 during competition assays or monoculture. Dotplots showing CFU of bacterial strains. Copper sulphate concentrations [in mM] are shown on the x-axis and Log10 CFU for *Salmonella* and *E. coli* are shown on the y-axis. Geom_smooth function from ggplot was used to draw a line between available datapoints and a 0.95 confidence interval was plotted (grey). Different colours correspond to CFU of strains in monoculture and co-culture (“competition”) and are shown on the legend attached below the figure. The limit of detection is shown as a grey dotted line

Our farm study demonstrated a decrease in the relative abundance of several commonly recognised probiotic/beneficial microbes in piglets fed a high-copper diet. Given that previous research has shown *S*. Typhimurium strains compete with *E. coli* both in vivo and in vitro [25, 59, 86], we investigated the interactions between selected porcine *Escherichia* isolates and *Salmonella*, specifically examining the influence of copper supplementation on these inter-species dynamics. We determined the impact of co-culture of 20 diverse *Escherichia* isolates, selected as described in Material and methods, on bioluminescent *S.* Typhimurium ST34 growth during anaerobiosis, to model the conditions of the host colon. Niche competition and niche invasion assays were used to model direct competition or the establishment of *Salmonella* colonisation when *E. coli* is already present in the colon. Initially, we investigated the interaction in the absence of copper supplementation to identify strains that competed better than others (Fig. 5A). Both assays showed that the addition of *E. coli* decreased the luminescence signal from *Salmonella* by at least 50% for 18 of the 20 *E. coli* strains tested (Fig. 5B), consistent with competition. Few pig *E. coli* isolates achieved similar levels of inhibition as the addition of *E. coli* Nissle 1917, a probiotic strain, previously proven to compete with various pathogenic bacteria. Further, some strains differed in their relative effectiveness at inhibiting *Salmonella* in niche competition or niche invasion. Four *E. coli* strains were selected for niche competition and niche invasion assays with the presence of copper sulphate based on the highest inhibitory activity, two with and two lacking the *sil*/*pco* gene cluster. All *E. coli* strains tested could significantly reduce *Salmonella* CFU in competition and invasion assays in medium without copper supplementation (Wilcoxon test, *p* < 0.05, Fig. S10). The addition of copper sulphate resulted in significant changes in the interaction trajectories between *Salmonella* and *E. coli* in niche competition assays, which was reflected by altered competitive indices (CIs) in media containing from 1 to 14 mM CuSO_4_ (Wilcoxon test, *p* < 0.05) (Fig. 5C) and the observation that *E. coli* was no longer able to reduce *Salmonella* CFUs in most of the tested concentrations (Fig. S10). Similar tendencies were observed for niche invasion assays, with the exception of the copper-resistant strain HCP6S3_I2, which inhibited *Salmonella* growth at five different (1, 5, 7, 8, and 9 mM) copper concentrations (Wilcoxon test, *p* < 0.05) (Fig. S10). Surprisingly, in the case of strains HCP4S3_I4, LCP10S3_I1, and LCP29S3_I5, an increase of *Salmonella* CFU was observed at two different copper concentrations (Fig. S10). The presence of copper resistance genes was key for *E. coli* survival during competition and invasion assays, but *Salmonella* dominated the copper-altered niche irrespective of *sil/pco* presence in *E. coli* (Wilcoxon test, *p* < 0.05) (Fig. 5C). Comparison of CFUs for copper-resistant *E. coli* strains (HCP4S3_I4, HCP6S3_I2) during niche competition assay and monoculture revealed two–four log10(CFU) reduction when *Salmonella* was present in the medium with copper (Fig. 5D). Taken together, our observations were consistent with the idea that copper sulphate substantially changes the interactions between *Salmonella* and *E. coli* in niche competition and invasion assays in vitro.

### Contact-dependent factors provide *Salmonella* significant advantage over *Escherichia* in the presence of therapeutic levels of copper

**Fig. 6:**
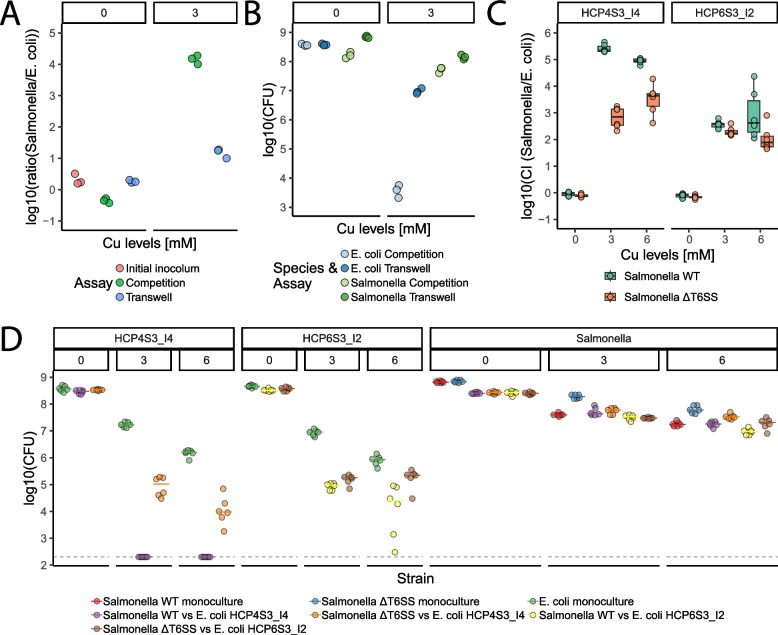
Contact-dependent competition between *Salmonella* and *E. coli* in copper sulphate-altered niche. **A** Ratios between *Salmonella* and *E. coli* HCP4S3_I4 in competition and Transwell assay with copper supplementation. Copper sulphate concentrations [in mM] are shown on the top x-axis. Log10 ratios between *Salmonella* and *E. coli* CFUs are shown on the y-axis, and the assay type and timepoint are marked with colours shown on the legend below the plot. **B** CFU counts for *Salmonella* and *E. coli* HCP4S3_I4 in competition and Transwell assay with copper supplementation. Copper sulphate concentrations [in mM] are shown on the top x-axis. Log10 CFUs for *Salmonella* and *E. coli* are shown on the y-axis, and the species and assay type are marked with colours shown on the legend below the plot. **C** Competitive index between *Salmonella* WT or ΔT6SS and *E. coli* strains in competition assay with copper supplementation. Copper sulphate concentrations [in mM] are shown on the bottom x-axis and *E. coli* strain name on the top x-axis. Log10 competitive index (CI) between *Salmonella* and *E. coli* CFUs is shown on the y-axis, and the *Salmonella* strain used for competition is marked with colours shown on the legend below the plot. **D** CFU counts for *Salmonella* and *E. coli* strains in monoculture or competition assay with copper supplementation. Genus or *E. coli* strain name and copper sulphate concentrations [in mM] are shown on the upper top x-axis and on copper sulphate concentrations (0, 3, 6 [mM]) in the media on the lower top x-axis. CFU of *E. coli* and *Salmonella* in competition or monocultures are shown on the y-axis, and the bacterial strain(s) is(are) marked with colours shown on the legend below the plot. Limit of detection is shown with a grey dashed line

Bacteria have evolved a wide range of mechanisms to compete with each other [38]. To determine how *Salmonella* outcompetes porcine *E. coli* isolates in the presence of copper, we first investigated if *S.* Typhimurium encodes bacteriocins that inhibit *E. coli* growth. No bacteriocins/antimicrobial peptides were found in the *Salmonella* genome using in silico approaches. Furthermore, an overlay disc diffusion assay of *S.* Typhimurium ST34 strain B54_C9 with *E. coli* strain HCP4S3_I4 revealed no growth inhibition of HCP4S3_I4 (data not shown). Next, the possibility of contact-dependent killing of *E. coli* by *S.* Typhimurium was tested using a Transwell system, which separates two bacterial populations with semi-permeable membranes with 0.45-µM pores. When the interaction of *Salmonella* and *E. coli* was compared in medium without copper supplementation, competition between both species was diminished in the Transwell system, which was reflected by an increase of the ratio *Salmonella*: *E. coli* (*t*-test, *p* < 0.001) and an increase of *Salmonella* CFU counts (*t*-test, *p* < 0.001) (Fig. 6A, B). Assays in medium with 3 mM copper supplementation revealed a major decrease of the ratio *Salmonella*: *E. coli* (*t*-test, *p* < 0.00005) and an increase in *E. coli* CFU counts in the Transwell system (*t*-test, *p* < 0.05), which indicated that contact-dependent interaction contributes to a great extent to the copper-induced *E. coli* killing by *Salmonella*.

As the Type 6 Secretion System (T6SS) was previously shown to be an important factor in competition between *Salmonella* and *Klebsiella* [84], the whole T6SS was disrupted in *S*. Typhimurium ST34 to investigate the contribution of this apparatus to the observed phenomena between *Salmonella* and two *E. coli* strains. Competition assays with the newly constructed strain revealed that the advantage of *Salmonella* over both tested *E. coli* strains in copper-supplemented media decreased significantly, which was reflected by lower CI values for ΔT6SS/*E. coli* when compared to wild-type *Salmonella*/*E. coli* (Wilcoxon test, *p* < 0.05) (Fig. 6C). Assays performed without copper supplementation showed no difference between CIs including wild-type *Salmonella* or ΔT6SS. Significant differences were found when CFUs of both *E. coli* strains were compared between co-incubations with wild-type *Salmonella* and ΔT6SS in 3 or 6 mM copper (Wilcoxon test, *p* < 0.05) (Fig. 6D). Small changes in CFUs were also observed after the competition of HCP4S3_I4 strain with wild-type *Salmonella* and ΔT6SS in medium without copper supplementation (Wilcoxon test, *p* < 0.05) (Fig. 6D). *Salmonella* ΔT6SS CFUs increased while co-incubated with *E. coli* in comparison to WT *Salmonella* co-incubated with the same *E. coli* strain at 6 mM (Wilcoxon test, *p* < 0.05) (Fig. 6D). Furthermore, *Salmonella* ΔT6SS CFUs increased in monoculture in comparison to WT *Salmonella* in monoculture at 3 and 6 mM (Wilcoxon test, *p* < 0.05) (Fig. 6D).

## Discussion

Foodborne pathogens remain one of the major causes of human infections worldwide [43]. Some of them, including *S*. Typhimurium, are also a significant cause of disease and reduced productivity in livestock. In such cases, disease prevention and management in affected animals can help reduce pathogen transmission to humans as well as boost livestock productivity. Several husbandry methods have been applied to increase the health and productivity of livestock, including feed additives to improve productivity through growth promotion, and have been shown to improve gut health and exclude pathogens. Since the turn of the century, changes in regulations necessitated a reduced reliance on antibiotics as growth promotors in animal production, leading to the common use of copper compounds in animal feed due to a lack of other alternatives [77].

Since the first report about the beneficial effects of copper sulphate on pig health and growth more than 60 years ago [4], many studies have reported this effect in farm studies. The most recent study was performed prior to or shortly following the emergence and global spread of copper-resistant *S*. Typhimurium ST34 [75], raising the question as to whether copper supplementation remains beneficial.

It has been known for some time that the beneficial effects of copper sulphate supplementation for pigs coincide with changes in intestinal microbiota [14, 37]. To further explore the changes in the microbial communities with greater resolution, we employed shotgun metagenomics to assess the effect of copper sulphate on weaned piglet microbiota. Consistent with previous studies [11, 107], no major changes in alpha and beta diversity indices were observed between piglets supplemented with therapeutic (150 ppm) and nutritional (10 ppm) levels of copper. The relative abundance of bacterial species belonging to *Bifidobacterium* and *Lactobacillus* decreased in piglets on a high copper sulphate diet in agreement with a previous report [11]. Increased abundance of bacteria belonging to the family Lachnospiraceae has been observed previously [33]; we made concordant observations for a previously unreported species of genus *Agathobacter* of the family Lachnospiraceae. The same study reported that bacteria from genus *Holdemanella* increased in relative abundance, which was not observed in our study. No differences in relative abundance of Enterobacteriaceae or *Escherichia* were reported in the study of [33], and [11] do not report any information about this family or genus [11, 33]. Several culture-based studies focused on changes in coliforms upon high copper sulphate supplementation in piglets, but the results were inconsistent, suggesting that there may be other factors affecting changes in coliforms/Enterobacteriaceae in these studies [48], leaving inconclusive results from community-sequencing based approaches.

Extensive culturing of gut bacteria has emerged in recent years as an approach to better characterise the functional attributes of the intestinal microbiota [12, 34]. We aimed to culture weaned piglet microbiota to investigate the resistance of microbes to copper sulphate. Previous studies focused on microbiota isolations from pigs using various culturing media and resulted in isolation of 110 species across nine phyla, including 22 novel species [102], 46 species from four phyla [31], 23 species from four phyla [66], and 148 species from 12 phyla with potentially four novel species [98]. In our approach, we isolated 131 species from five phyla, including 39 species taxa that could not be assigned to known species using current databases and are therefore candidate new species. This indicated that despite recent advances in pig gut microbiota culturomics, a large portion of uncultured microbiota remains to be explored in future studies.

Cultured strains of species from the genera *Bulleidia*, *Bifidobacterium*, *Limosilactobacillus*, *Lactobacillus*, and *Prevotella* had a relatively high susceptibility to copper sulphate MIC in vitro, even compared to *E. coli* and *S.* Typhimurium ST34 that lack copper resistance genes *sil*/*pco*. This was consistent with an observed decrease in relative abundance of species belonging to these genera observed using metagenomics. Surprisingly, despite the reduction in relative abundance of *E. coli* in piglets on a high-copper diet in our metagenomics data, we observed commonly occurring strains that exhibited a high level of resistance to copper sulphate. These results indicate that reduction of *E. coli* abundance might not be a direct effect of copper toxicity. Instead, this may be due to indirect effects of copper toxicity on the microbiota community that affect the niche or inter-bacterial interactions. In vitro niche competition assays (Fig. 5D) indicated that copper-resistant *E. coli* in monoculture with copper sulphate supplementation grew to lower CFUs than *Salmonella*, indicative of different adaptation mechanisms to stress imposed on *sil*/*pco*-positive *E. coli* by copper. Furthermore, the presence of copper sulphate as a stressor induced competition mechanisms between *Salmonella* and *E. coli*, decreasing the survival of *E. coli*. It has been shown previously that several bacterial species induce toxin production or activate T6SS upon exposure to various stresses. It is therefore possible that species of the gut microbiota also elaborate killing mechanisms in the gut of piglets with high copper supplementation [23, 39]. Alternatively, copper sulphate may alter the host gut environment, impacting oxygen levels and thereby reducing *E. coli* relative abundance. Limited oxygen is known to decrease the abundance of facultative anaerobes like *E. coli* [65], and oxygen availability has been linked to host epithelial metabolism and inflammation [15]. Considering that high copper supplementation has been associated with increased epithelial villus height, reduced crypt depth, reduced intestinal permeability, and inflammation [27, 108], the direct effect of copper sulphate on host cells should be considered in the future as a possible factor affecting microbiota assembly and *E. coli* relative abundance [22].

Since the phylum Pseudomonadota (dominated by *Escherichia* in our study) has been identified as a group with the highest number of copper resistance genes associated with increased copper sulphate MIC, we investigated the genetic background of this phenotype. We were able to detect *sil* together with *pco* genes as well as *sil* genes alone encoded on the chromosome or on a plasmid of *E. coli* strains isolated from piglets, which corroborates previous reports where copper resistance cluster *sil*/*pco* has been identified in various Enterobacteriaceae on plasmids or chromosomes [10, 17].

The *sil* operon has been initially identified as a key genetic determinant providing resistance to silver, but *Salmonella* genomic island 4 (SGI-4) encoding *sil* has been confirmed as a major contributor to copper resistance in *S*. Typhimurium ST34 [10]. However, there are still aspects that remain unclear in other serovars or Enterobacterales species. While several studies, including ours, have established *sil* as the primary driver of copper resistance in *S*. Typhimurium in anaerobic conditions [10, 64], a recent investigation in *S*. Senftenberg suggests that *pco* can confer resistance as well [45]. Contribution of *sil*/*pco* to copper resistance in *E. coli* has been questioned in the study of [17], even though three out of four strains carrying these clusters had higher copper sulphate broth MIC than three *sil*/*pco*-negative strains [17]. The role of *pco* in providing copper resistance has been questioned, yet a possible contribution of *sil* to copper resistance was not considered [105]. Copper sulphate MIC determined by a broth dilution on *E. coli* isolated during our study (Fig. 5C) in anaerobic conditions revealed that different *sil*/*pco* variants provide similar levels of copper resistance, but all *Escherichia* isolates lacking *sil*/*pco* or *sil* clusters had an MIC at least 6 mM lower than *sil*/*pco*- or *sil*-positive *Escherichia*. Removal of *sil* or *sil*/*pco* from *S*. Typhimurium ST34 yielded a copper MIC like that of *S*. Typhimurium SL1344 (which has no copper resistance genes), and disruption of *pco* in ST34 resulted in an increase of MIC by 1 mM (Fig. 4C), defining *sil* and *pco* involvement in copper resistance. Our analysis revealed that the *sil/pco* gene clusters were often associated with mobile genetic elements (MGE) such as transposons or plasmids in the phylogenetically diverse *E. coli* strains being investigated, concordant with the proposed role of MGE in copper resistance spread [17, 29]. Plasmid-encoded *sil/pco* clusters in our collection co-localised with IS1- and IS3-like genetic elements, which, to our knowledge, was not previously reported, indicating that copper resistance-associated mobilome can be more diverse than previously expected.

As our metagenomic and culturing investigations indicated the impact of copper sulphate on microbiota composition, we aimed to investigate if therapeutic levels of copper sulphate can affect interactions between the common pig pathogen *S.* Typhimurium ST34 and porcine *E. coli* isolates. Several studies have identified bacteriocins as important factors in competitive interactions between these two species. For example, colicin Ib mediates competitive advantage for *S.* Typhimurium SL1344 in the inflamed gut [68], but we did not find any bacteriocin sequences in the genome of*S.* Typhimurium ST34 or any indications that copper sulphate induces bacteriocin production. Furthermore, it has been previously shown that *S.* Typhimurium ST34 isolates lack plasmids that typically encode bacteriocins, such as that present in SL1344 encoding for colicin Ib [6]. As Transwell assays revealed that the competitive advantage of *S.* Typhimurium ST34 over porcine *E. coli* isolates in copper-supplemented media is contact-dependent, we investigated the contribution of T6SS to these interactions, which revealed that indeed this system confers ST34 advantage over porcine *E. coli*. Previous research indicated that T6SS of *S.* Typhimurium LT2 can mediate the killing of laboratory *E. coli* strain K-12 W3110 [13]. Another report with the use of SL1344 and *E. coli* mouse commensal strain JB2 showed that the T6SS does not contribute to competition between these two strains [84]. Indeed, we observed considerable differences in the degree of T6SS-dependent killing of the two strains of *E. coli* by a *S*. Typhimurium ST34 strain. The reason for this is not known, and the two strains were highly divergent and therefore contained many insertions and deletions and single nucleotide polymorphisms that could account for this observation. Our results, together with previously published research, indicate that the contribution of T6SS to *E. coli* killing might be strain-dependent, but more work is needed to explain the mechanism of *E. coli* resistance against *Salmonella* T6SS.

The increase in T6SS-mediated killing of *E. coli* by copper sulphate poses the question as to how this compound activates expression of this secretion system or how copper sulphate increases susceptibility of *E. coli* to *Salmonella*’s T6SS. Ferric uptake regulator Fur is a pivotal global transcriptional regulator in bacteria, primarily recognised for its central role in maintaining iron homeostasis, but this protein has been shown to repress T6SS in *S.* Typhimurium in iron-rich media [97]. Although the link between iron and copper homeostasis has not been studied previously in *Salmonella*, several reports show cross-talk between iron and copper homeostasis in *E. coli* [47]. Study of [49] reported increased expression of *fur* and enterobactin operon in *E. coli* upon high (2 mM) copper exposure in aerobic conditions [49]. Deletion of periplasmic multicopper oxidase (CueO) increased intracellular levels of iron in uropathogenic *E. coli*, which indicated that this protein is critical not only for copper homeostasis, but iron as well [96]. As multiple other factors have been implicated in T6SS regulation in various microbes [44], the role of copper sulphate as T6SS activator requires further research.

Our study is consistent with the view that high copper supplementation in weaned piglets selectively impacts the relative abundance of gut microbiota, affecting key members associated with microbiome function. The increased presence of copper resistance genes in Pseudomonadota, particularly the acquisition of *sil/pco* clusters, directly enhances copper resistance in *E. coli*. This resistance is a necessary, but not sufficient, factor for competitive interactions between *Salmonella* and *E. coli* under high-copper conditions in vitro. *Salmonella* possesses additional mechanisms like T6SS that provide a competitive advantage in this copper-rich environment. The prevalence of copper-resistant *Salmonella* Typhimurium ST34 in pigs [76] and diverse *E. coli* found in these studies raises important questions. First, is the use of therapeutic levels of copper in pig feed still beneficial in boosting gut health and productivity in pigs. Second, whether *Salmonella* can exploit the altered gut niche resulting from copper supplementation, and if this has a negative impact on the risk to food safety.

## Material and methods

### Farm study

**Fig. 7:**
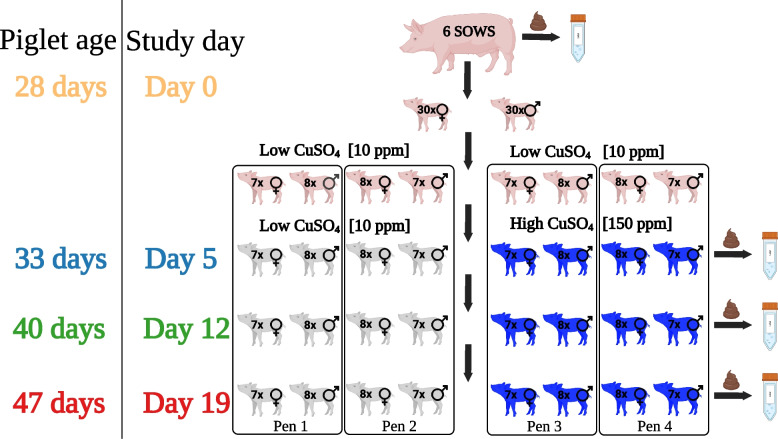
Farm study design and sample collection. Sixty commercial piglets from six sows were divided into four pens. Half received a high copper (150 ppm Cu) starter diet, and the other half received a low copper (10 ppm Cu) starter diet. Fresh faecal samples were collected on days 5, 12, and 19 and stored at −80 °C

Farm study was performed on a commercial farm and utilised 60 commercial piglets derived from six sows (Fig. 7). Sows were kept on a commercial low-copper diet. Piglets were separated into four pens with similar numbers, sex, and weight of piglets in each of the pens. All piglets were kept on a commercial pre-starter low-copper diet. After 5 days, 30 piglets were put on the high copper (150 ppm Cu) commercial starter diet and the rest were kept on the low copper (10 ppm Cu) starter diet. Freshly voided faecal samples were collected from piglets at days 5, 12, and 19 and immediately placed into 50-ml falcon tubes and kept on ice for 2–6 h. Faecal samples were aliquoted in anaerobic conditions (Whitley A35 Workstation) for storage frozen at −80 °C. The pig study received a favourable ethical opinion from the University of Surrey’s Non-Animals Scientific Procedures Act (NASPA) ethics committee (NASPA-2122-07).

### Shotgun metagenomic sequencing and analysis

DNA from faecal samples was isolated using a Maxwell RSC 48 Instrument and Maxwell RSC PureFood GMO and Authentication kit with minor modifications. Briefly, 1 ml of CTAB buffer and around 100 mg of faeces were added to a lysing matrix E tube (MP Biomedicals). Samples were heated at 95 °C for 5 min, vortexed, and homogenised in the FastPrep24 Instrument for 45 s at a frequency of 6.0 m/s. Next, 40 µl of Proteinase K and 20 µl of RNase A were added; samples were vortexed and incubated at 70 °C for 10 min. Three hundred microlitres of the lysates was used for purification with Maxwell RSC 48 Instrument. DNA concentration was measured with Qubit Fluorometer and Qubit™ dsDNA BR Quantification Assay Kit. DNA was diluted to 5 ng/µl, and library was prepared using tagmentation protocol (Illumina). The libraries were quantified using the Promega QuantiFluor® dsDNA System (Catalogue No. E2670) and run on a GloMax® Discover Microplate Reader. Libraries were pooled following quantification in equal quantities. The final pool was double-SPRI size selected between 0.5 and 0.7× bead volumes using sample purification beads (Illumina® DNA Prep, (M) Tagmentation (96 Samples, IPB), 20060059). The final pool was quantified on a Qubit 3.0 instrument and run on a D5000 ScreenTape (Agilent Catalogue No. 5067-5579) using the Agilent Tapestation 4200 to calculate the final library pool molarity. The pool was sent to Source Bioscience and run on two NovaSeq S4 lanes. Two hundred ninety-five pig metagenomic samples from BioProject PRJEB11755 were downloaded for additional analyses [103].

MG-TK pipeline was used for read quality assessment, metagenome-assembled genomes assembly, read mapping, and species abundance determination [35]. Host reads were filtered out by using Kraken v2.1.0 with parameter “--confidence 0.01” and database generated with *Sus scrofa* genomes assembly (GCF_000003025.6) [99, 100]. Raw shotgun metagenomes were quality filtered using sdm v1.63 with default parameters [71], assembled using MEGAHIT v 1.2.9 with parameters “--k-list 25,43,67,87,101,127” [57], and reads mapped onto assemblies using Bowtie2 v2.3.4.1 with parameters “--end-to-end” [56], genes predicted with Prodigal v2.6.1 with parameters “-p meta” [46], and a gene catalogue clustered at 95% nt identity using MMseqs2.80 [63]. Metagenomic assembled genomes (MAGs) were binned using SeminBin2 [72] and combined with canopy clusters [69]in MG-TK to species-level dereplicated MGS (metagenomic species). RTK was used to calculate abundance matrices from MGS representative genes in the gene catalogue [83]. Vegan package was used for analysis of alpha (Shannon and Simpson’s indices) and beta (Brey-Curtis dissimilarity) diversity in microbiota (Oksanen [70]. Anosim function with 1000 permutations from vegan package was used to test for difference in beta diversity between groups [89]. Wilcoxon rank-sum test with Benjamini-Hochberg correction for multiple comparisons was used to compare differences in relative abundance between microbiota [58].

### Culturing of bacterial isolates

Faecal samples from 6 piglets (3 from high and low copper supplementation) and 1 sow were used for isolation of anaerobic bacteria in an anaerobic cabinet using pre-reduced reagents following the protocol of [12], with minor changes [12]. Frozen faecal samples were diluted in PBS to a concentration of 0.1 g/ml of PBS and serially diluted on YCFA agar plates (2 × 90 mm for each dilution) supplemented with 0.002 g/ml each of glucose, maltose, and cellobiose or starch. For a subset of samples, 0.1 g of faeces was treated with 70% ethanol for 4 h at room temperature, washed three times with PBS, and serial dilutions plated on YCFA agar supplemented with 0.1% sodium taurocholate to isolate ethanol-resistant endospores (spores). Ninety-five colonies were picked for each sample after 72 h from plating in an anaerobic cabinet. Each colony was purified by re-streaking and culturing on YCFA agar 3 times. A single colony was then selected from the last plate and used for YCFA broth culture and cryo-stocks. If bacteria did not grow in YCFA broth for 1 week, 2–3 YCFA plates were used to spread a single colony, and bacteria were harvested from the plates after 3 days with the aid of 2 ml of YCFA and an L-shaped inoculating loop and used for cryo-stocks and genomic DNA isolation. Bacterial cryo-stocks were generated by mixing an equal amount of bacterial culture with 40% glycerol in ddH_2_O. Seven hundred fifty microlitres of bacterial culture was used for DNA isolation. Cultures were centrifuged 15,000×g for 5 min, washed in PBS, and pellets were frozen for at least 24 h at −80 °C.

Faecal samples from 28 piglets (14 from high and low copper supplementation) and 6 sows were used for isolation of *Enterobacteriaceae* in aerobic conditions. Frozen faecal samples were diluted in PBS to a concentration of 0.1 g/ml of PBS and serially diluted on Eosin-Methylene Blue (EMB) agar plates (90 mm, 1 plate per dilution) and incubated at 37 °C for 24 h. Five single colonies were re-streaked onto MacConkey agar and subsequently onto LB agar (10 g tryptone, 5 g yeast extract, 10 g NaCl, 15 g agar per litre). Subsequently, each isolate was cultured aerobically O/N in 5-ml LB broth (10 g tryptone, 5 g yeast extract, 10 g NaCl per litre), and O/N cultures were used for DNA isolation and cryostock preparation.

DNA from bacteria was isolated with use of Maxwell RSC 48 Instrument and Maxwell RSC PureFood GMO and Authentication kit with slight modifications. Frozen bacterial pellets were resuspended in 500 µl of CTAB buffer with chicken lysozyme (30 mg/ml) and incubated at 37 °C for 1 h. Next, 30 µl of Proteinase K and 20 µl of RNase A were added; samples were vortexed and incubated at 60 °C for at least 1 h, aerobically. Three hundred microlitres of the lysates was used for purification with Maxwell RSC 48 Instrument. DNA concentration was measured with Qubit Fluorometer and Qubit™ dsDNA BR Quantification Assay Kit. Library preparation protocol was the same as the one used for metagenomic samples. Sequencing was performed using Novaseq500 or NextSeq2000. Genome assembly and annotation was performed with shovill pipeline v1.1.0 and Bakta v1.5.0, respectively [53, 87]. Proteins annotated with Bakta as copper homeostasis genes were pooled together and redundant genes were dereplicated with CD-HIT v4.8.1 with parameters “-c 0.8 -s 0.8” and annotated with InterPro database [36, 74]. Proteins with no domains associated with copper/heavy metal homeostasis/resistance were removed and used for blastp query of all bacterial genomes (coverage and identity of 80% and above were considered a positive hit). CheckM v1.2.1 was used for contamination control and GTDB-Tk v2.1.1 with database version 214 was used for species determination [21, 73]. Quast v4.6.3 was used for assembly statistics [41]. Genomes of bacterial isolates with no species name assigned by GTDB-tk were analysed with TYGS platform [62]. Comparison of cultured microbiota with species present in metagenomic samples was done with dataset generated during this study, dataset of 295 pig metagenomic samples from France, Denmark, and China and all pig dataset analysed with Pig Gut v1.0 MGnify Genome database accessed on 21.02.2024 [40, 103]. All shotgun metagenomic data was additionally analysed with metaphlan v.4.1.1 and database mpa_vJun23_CHOCOPhlAnSGB_202403 [60].

### *Escherichia* genomics

ClermonTyping was utilised for strain phylotyping [8]. Abricate with a custom database containing: (1) SGI-4 coding sequences (CDSs); (2) *E. coli* copper homeostasis genes; or (3) genes encoding aerobactin and yersiniabactin was used to determine the presence of these genes in *E. coli* assemblies isolated from pigs [91]. Snippy v4.6.0 was used for reference-based phylogeny, and *E. coli* MG1655 (NC_000913.3) was used as a reference for mapping and SNP-calling (Seeman [88]. RAxML-NG v1.1 with GTR + G model and 1000 bootstrap replicates was used for phylogenetic tree calculation [55]. R packages ggtree and ggtreeExtra have been utilised for phylogenetic tree annotation [104, 106]. BLAST version 2.12 was used for*sil* or *pco* cluster extraction from *E. coli* genomes [1]. *Sil* or *pco* gene clusters were aligned with Clustal Omega, and the tree was generated with RAxML-NG [92]. A distance matrix based on SNP-distance was generated with snp-dist included in Snippy software. *Sil* and *pco* gene cluster was considered a new variant if the distance to the closest variant was more than 10 and 3 SNPs, respectively.

### Bacterial strains and mutants

*Salmonella* mutants were generated by exchange of the region of interest (gene, gene cluster) with kanamycin (*aph*) or chloramphenicol (*cat*) cassette tagged with 50 bp extensions homologous to regions overlapping the DNA fragment being replaced [24]. To minimise the potential for polar effects on surrounding genes, primers were designed to precisely remove the entire *sil* and *pco*loci that are known to form a commonly mobilised region that together function in resistance to copper. For deletion of the T6SS SPI-6 gene cluster, to minimise potential off-target polar effects, we deleted the same genomic region that was previously used for which functional loss was complemented by the T6SS SPI-6 gene cluster encoded on a plasmid [78, 84], indicating that the deletion did not have polar effects on neighbouring genes. Phage Lambda homologous recombination machinery genes encoded on the pSIM18 plasmid were used to induce homologous recombination, and antibiotic cassette insertion allowed selection of strains devoid of the region of interest [18]. Correct gene/gene cluster removal was confirmed with colony PCR. Whole genome sequencing was used to confirm the lack of off-target mutations of new strains [50].

Nalidixic acid spontaneous mutants of *E. coli* were selected by plating O/N broth cultures on increasing concentrations of antibiotic (0, 10, 25, 50, 100, 200 µg/ml). Strains that grew on the highest nalidixic acid concentrations were selected and submitted to whole genome sequencing. The presence of mutations in nalidixic acid resistant strains was investigated by mapping raw reads of wild type and mutants to the assembly of wild-type strain and determining SNPs and indels using the snippy pipeline (Seeman [88]. All SNPs detected only in mutants were confirmed by visualisation of BAM files in ARTEMIS software [16]. Growth curve experiments were performed to investigate the influence of nalidixic acid mutation on strain growth abilities [52]. Briefly, overnight cultures were diluted to 5 × 10^6^ bacteria/ml in LB, and 150 µl bacterial solution was pipetted into each well of a 96-well plate. Bacterial growth was measured with Logphase600 (BioTek) at 37 °C for 24 h with constant shaking (700 rpm) and OD_600_ measurements every 10 min. Wild type and mutants were included in each experiment. At least three biological replicates with at least four technical replicates were performed for each strain (Fig. S11). One strain with a single mutation in the *gyrA* gene, no off-target mutations, and no growth defect in LB was selected for experimental procedures.

Luminescent *Salmonella* B54 WT strain was constructed by integrating *lux*operon into 16S site [80]. Briefly, p16Slux plasmid was transformed into *Salmonella*, and heat stress (42 °C, 24 h) was used to induce integration of *luxABCDE* operon into *ssu* locus, which was confirmed by colony PCR. Growth curves and WGS were utilised to select a strain with a similar growth rate to WT and no off-target mutations, respectively.

All strains are listed in Table 1. All primers are listed in Table 2.

**Table 1:** Bacterial strains used in this study

Strain	Relevant feature(s)	Reference
*S.* Typhimurium ST34 B54-C9	Wild type	This study
*S.* Typhimurium B54_C9Δ*sil*-*pco*::*kan*−1	*sil*/*pco* cluster deletion mutant	This study
*S.* Typhimurium B54_C9Δ*sil*::*kan*−1	*sil* cluster deletion mutant	This study
*S.* Typhimurium B54_C9Δ*pco*::*kan*−1	*pco* cluster deletion mutant	This study
*S.* Typhimurium B54_C9_SGI4mark1::*kan*−1	Insertion of kanamycin resistance into SGI-4	This study
*S.* Typhimurium B54_C9::p16Slux-1	Integration of *lux* cassette into 16S region	This study
LCP10S3_I1_NalR1	Nalidixic acid resistant	This study
LCP29S3_I5_NalR4	Nalidixic acid resistant	This study
HCP4S3_I4_NalR1	Nalidixic acid resistant	This study
HCP6S3_I2_NalR2	Nalidixic acid resistant	This study
*S.* Typhimurium B54_C9ΔT6SS::*kan*−1	T6SS cluster deletion mutant	This study

**Table 2:** Primers used in this study

ID	Name	Sequence (5'-3')	Reference
QRK006	k1	CAGTCATAGCCGAATAGCCT	[24]
QRK007	k2	CGGTGCCCTGAATGAACTGC	[24]
QRK008	c1	TTATACGCAAGGCGACAAGG	[24]
QRK009	c2	GATCTTCCGTCACAGGTAGG	[24]
QRK010	silEdelfor	AGGAATAATCTATCAAGGAAAGGGTAAAAGCACGGATACTACAGTCGCATGTGTAGGCTGGAGCTGCTTC	This study
QRK011	pcoEdelrev	AAACCAGTGATGCCAGCGTCAAAAGAGGGTCTAACAAATGGGGCTGCGGGCATATGAATATCCTCCTTAG	This study
QRK012	silE100UpFor	TACCGGTTAATTGTAGCTGAGTC	This study
QRK013	silEinternalRev	ATGAAACCATGACGAACGGA	This study
QRK014	pcoE100DownRev	GAACACTCACACTGTCACCC	This study
QRK015	silPdelrev	TACTTTTCATACTGGACTCCTTCTGTTCGTAACAGACCCTTCACTCAGAGCATATGAATATCCTCCTTAG	This study
QRK016	silP100DownRev	GGGCAGACCAGCAATAACA	This study
QRK017	pcoGdelfor	ACGATAAAAAAAATTAATTCGGCAAACGGGGCCGCGTCGCGGTCCCGTTAGTGTAGGCTGGAGCTGCTTC	This study
QRK018	pcoG100UpFor	TGTTATTGCTGGTCTGCCC	This study
QRK019	pcoGinternalRev	CCCGGACCGAATACAACAG	This study
QRK073	SGI4mark1delfor	AGTACACAATAAAAAAACCCGAAGTAAATCGGGTTTTAATTATTTAACGTGTGTAGGCTGGAGCTGCTTC	This study
QRK074	SGI4mark1delrev	AACGCCATGATAAGCGTACTTTTAAATCACTCCCGGGCACGGGAGCCTGTCATATGAATATCCTCCTTAG	This study
QRK075	SGI4mark1.100UpFor	CAACCTAACATGAAGGAACACAGG	This study
QRK076	SGI4mark1.100DownRev	GCAATGGCTGAAACCGAGC	This study
QRK107	16S_rev_XhoI	CTGATCTCGAGGGCGGTGTGTACAAGG	[80]
QRK108	16S_fwd_int	ATTAGCTAGTAGGTGGGGTAACGGCTCACCTAGG	[80]
QRK123	T6SSdelFor	TTTTTATACATCCTGTGAAGTAAAAAAAACCGTATCACTGTAAAAGGGATGTGTAGGCTGGAGCTGCTTC	This study
QRK124	T6SSdelRev	ATGGCACATTAATTTGAAGCAGCTCTCATCCGGTATCGCTTTTCAGTGCACATATGAATATCCTCCTTAG	This study
QRK125	T6SS100UpFor	GCAGCAACTGATTCAAAAGGTGAG	This study
QRK126	T6SS100DownRev	GTCTCAACACTAAGAGCTGACTGA	This study
QRK127	T6SSinternalRev	GGGATCAAAATAGCCATGACAGTG	This study

### Copper minimal inhibitory concentrations assays

Minimal inhibitory concentration screen for pig microbiota (anaerobic bacteria and 100 *E. coli* isolates) was performed by spotting bacterial suspensions onto YCFA agar plates supplemented with increasing concentrations of CuSO_4_ (concentrations tested: 0, 0.156, 0.313, 0.625, 1.25, 2.5, 5, 10, 20 [mM]). Isolate selection criteria were as follows: (1) for bacteria cultured on YCFA: at least one isolate for each species isolated and in case when more isolates were cultured, phylogeny, snp-distance, and pangenome were taken into account during strain selection and (2) for bacteria cultured on EMB and MacConkey: 100 isolates. Anaerobic bacteria were grown on YCFA agar plates (1 strain per plate) for 3 days, re-streaked on YCFA agar for another 3 days at 37 °C, and scraped into PBS. *E. coli* isolates were grown O/N in a 96-well plate in LB broth. Five microlitres of bacterial suspension was spotted onto YCFA agar and incubated for 3 days in anaerobic conditions. MIC was defined as the lowest concentration where bacterial growth was not observed on plates for two technical replicates. *Salmonella* WT and Δ*sil-pco*::*kan*−1 were included in all experiments as reference.

Minimal inhibitory concentrations (MIC) for *Salmonella* strains and selected *E. coli* strains were performed using the broth microdilution method [10]. *E. coli* strains were selected based on phylogroup, *sil*/*sil-pco* cluster presence, and *sil*/*sil-pco* cluster variation (Table S3). Bacteria were grown in anaerobic conditions in 5-ml LB broth, diluted in LB in 25 mM HEPES buffer (pH = 7.4, later referred to as “LB HEPES”) to 1 × 10^6^ CFU/ml, and 100 µl was inoculated into 100 µl of serial dilution of CuSO_4_ (2 to 40 in 2 mM intervals) in LB HEPES, allowing for MIC testing in the range from 0 to 20 mM in 1-mM intervals in a 96-well plate. Optical density of bacteria incubated in anaerobic conditions for 24 h was measured at 600 nm with a BMG OMEGA plate reader. The MIC was defined as the lowest concentration where bacterial growth was not observed for at least three biological replicates.

### Ecological niche competition and invasion assays

A screen of the interactions between *Salmonella* and *Escherichia coli* pig isolates was performed with use of *lux*-tagged *Salmonella* and 20 pre-selected *E. coli* isolates (Table S3). Bacteria were grown in 5-ml LB broth in anaerobic conditions overnight. *Salmonella* was diluted in LB to 5 × 10^7^ CFU/ml and 20 µl was used for niche competition and invasion assays. In the case of *E. coli*, 20 µl or 180 µl of O/N culture was used for niche competition and invasion assays, respectively [93]. Only the *Salmonella* control consisted of 20 µl of diluted bacteria and 180 µl of LB. Bacterial isolates were incubated in 96-well plates for 24 h in anaerobic conditions and then transferred in aerobic conditions to a white polypropylene 96-well plate. Luminescence was measured using a BMG OMEGA plate reader. The experiment was performed in at least three technical and biological replicates.

*S.* Typhimurium B54-C9 and *E. coli* strains with nalidixic acid resistance were grown in 5-ml of LB HEPES O/N in anaerobic conditions for niche competition and invasion assays in CuSO_4_-containing media. LB HEPES with increasing concentrations of CuSO_4_ was used in the assays. 1 × 10^5^ CFU of *Salmonella* and *E. coli* was used for the competition assay in 200 µl of LB HEPES. Controls included only 1 × 10^5^ CFU of a single strain incubated in the same conditions. 1 × 10^5^ CFU of *Salmonella* and 50 µl of *E. coli* O/N cultures were used for the invasion assay in a total volume of 200 µl of LB HEPES. To confirm the initial inoculum for each strain, bacterial dilutions were plated on selective media and CFU counted the next day. The assay lasted for 24 h and CFU counts were determined by serial dilution plating on selective media. The experiments were performed in at least four biological replicates.

Bacteriocin production was tested using an overlay disc diffusion assay [51]. O/N cultures of *S.* Typhimurium B54_C9 and *E. coli* HCP4S3_I4 were grown in anaerobic conditions in LB HEPES supplemented with 0 or 3 mM CuSO_4_ at 37 °C. One hundred microlitres of O/N *E. coli* culture was added to melted soft LB agar (0.5% agar) supplemented with 0 or 3 mM CuSO_4_, mixed and overlaid over an LB agar plate. Whatman paper 5-mm discs were placed on solidified agar and 10 µl spots of *S.* Typhimurium B54_C9 O/N cultures. Plates were incubated O/N at 37 °C and checked for zones of inhibition on the following day. Macrel software was used to search *S.* Typhimurium B54_C9 genome for the presence of antimicrobial peptides [85]. Bactibase2 bacteriocin protein sequences and blastx were used on *S.* Typhimurium B54_C9 genome to search for bacteriocins [42].

Transwell competition assays were performed with the use of Corning™ Transwell™ 6 Well Plate with Permeable Polyester Membrane Inserts (0.4-µm pores) and LB HEPES without or with 3 mM CuSO_4_ [54]. *S.* Typhimurium B54-C9 and *E. coli* HCP4S3_I4_NalR1 were grown O/N in 5 ml of LB HEPES in anaerobic conditions. Bacteria were diluted in LB HEPES to 1 × 10^6^ CFU/ml. Next, 1.3 ml of LB HEPES or LB HEPES with 6 mM CuSO_4_ was added to Corning plates and universal tubes, followed by 1.3 ml of *E. coli*. Transwell inserts were placed in the Corning plate and 0.75 ml of LB HEPES or LB HEPES with 6 mM CuSO_4_ was added to inserts and universal tubes. Next, 0.75 ml of *Salmonella* was added to Transwell inserts and universal tubes. CFU counts were determined by serial dilution plating on selective media for initial inocula and bacteria incubated for 24 h.

## Supplementary Information

Supplementary Material 1. Dataset S1

Supplementary Material 2. Dataset S2

Supplementary Material 3. Figure S1-S11

Supplementary Material 4. Table S1

Supplementary Material 5. Table S2

Supplementary Material 6. Table S3

## Data Availability

Pig faecal shotgun metagenomics data for this study are freely available from the NCBI BioProject database under accession number PRJNA1219188. Data associated with pig microbiota culturing are available from the NCBI BioProject database under accession numbers: PRJNA1273087, PRJNA1273977 and PRJNA1276128.
