# Supplementary material for: Copper is an intestinal habitat filter affecting the gut microbiota interactions with Salmonella Typhimurium
Source: Microbiome. 2026 Mar 28;14:110. doi: 10.1186/s40168-025-02322-4 (PMC13064011; doi:10.1186/s40168-025-02322-4)
Supplement: Supplementary file 2 — Supplementary Material 1. Dataset S1 [file 40168_2025_2322_MOESM1_ESM.html]

Culturomics of porcine new bacterial species


# Culturomics of porcine new bacterial species

#### Rafal Kolenda

#### 2025-03-30

# 1 Culturomics:


## 1.1 Taxonomic classification

### 1.1.1 GTDB-Tk

#### 1.1.1.1 Table

#### 1.1.1.2 Genus/Species barplot

#### 1.1.1.3 Classification barplot

#### 1.1.1.4 Classification barplot with isolation source

## 1.2 New species analysis

### 1.2.1 Atopobiaceae\_Olsenella


#### 1.2.1.1 TYGS results

Identified as 3 potentially new species

##### 1.2.1.1.1 16 phylogeny

##### 1.2.1.1.2 digital DNA-DNA hybridization (dDDH)

### 1.2.2 Gracilibacillus


#### 1.2.2.1 TYGS results

Identified as 1 potentially new species

##### 1.2.2.1.1 16 phylogeny

##### 1.2.2.1.2 dDDH

### 1.2.3 Lactimicrobium

Identified as 1 potentially new species

##### 1.2.3.0.1 16 phylogeny

##### 1.2.3.0.2 dDDH

### 1.2.4 Coprococcus

Identified as 1 potentially new species

##### 1.2.4.0.1 16 phylogeny

##### 1.2.4.0.2 dDDH

### 1.2.5 Bilifractor

Identified as 1 potentially new species

##### 1.2.5.0.1 16 phylogeny

##### 1.2.5.0.2 dDDH

### 1.2.6 Blautia

Identified as 4 potentially new species

##### 1.2.6.0.1 16 phylogeny

##### 1.2.6.0.2 dDDH

### 1.2.7 CAG-791

Identified as 1 potentially new species

##### 1.2.7.0.1 16 phylogeny

##### 1.2.7.0.2 dDDH

### 1.2.8 Oribacterium

Identified as 1 potentially new species

##### 1.2.8.0.1 16 phylogeny

##### 1.2.8.0.2 dDDH

### 1.2.9 Paralachnospira

Identified as 1 potentially new species

##### 1.2.9.0.1 16 phylogeny

##### 1.2.9.0.2 dDDH

### 1.2.10 RUG115

Identified as 1 potentially new species

##### 1.2.10.0.1 16 phylogeny

##### 1.2.10.0.2 dDDH

### 1.2.11 Weimeria

Identified as 1 potentially new species

##### 1.2.11.0.1 16 phylogeny

##### 1.2.11.0.2 dDDH

### 1.2.12 UBA1417

Identified as 1 potentially new species

##### 1.2.12.0.1 16 phylogeny

##### 1.2.12.0.2 dDDH

### 1.2.13 CABMKH01

Identified as 1 potentially new species

##### 1.2.13.0.1 16 phylogeny

##### 1.2.13.0.2 dDDH

### 1.2.14 Luteococcus

Identified as 1 potentially new species

##### 1.2.14.0.1 16 phylogeny

##### 1.2.14.0.2 dDDH

### 1.2.15 Dysosmobacter

Identified as 1 potentially new species

##### 1.2.15.0.1 16 phylogeny

##### 1.2.15.0.2 dDDH

### 1.2.16 JAAZME01

Identified as 1 potentially new species

##### 1.2.16.0.1 16 phylogeny

##### 1.2.16.0.2 dDDH

### 1.2.17 Fundicoccus

Identified as 1 potentially new species

##### 1.2.17.0.1 16 phylogeny

##### 1.2.17.0.2 dDDH

### 1.2.18 Prevotella\_sp900546535

Identified as 1 potentially new species

##### 1.2.18.0.1 16 phylogeny

##### 1.2.18.0.2 dDDH

### 1.2.19 Blautia\_A\_sp934359835

Identified 1 potentially new species

##### 1.2.19.0.1 16 phylogeny

##### 1.2.19.0.2 dDDH

### 1.2.20 CAG\_964\_sp902789345

Identified as 1 potentially new species

##### 1.2.20.0.1 16 phylogeny

##### 1.2.20.0.2 dDDH

### 1.2.21 Clostridium\_sp012519155

Identified as 1 potentially new species

##### 1.2.21.0.1 16 phylogeny

##### 1.2.21.0.2 dDDH

### 1.2.22 Dysosmobacter\_sp022778785

Identified as 1 potentially new species

##### 1.2.22.0.1 16 phylogeny

##### 1.2.22.0.2 dDDH

### 1.2.23 Lawsonibacter

Identified as 2 potentially new species

5 isolates identified as separate species in GTDB did not give any
results in TYGS

##### 1.2.23.0.1 16 phylogeny

##### 1.2.23.0.2 whole genome phylogeny

##### 1.2.23.0.3 dDDH

### 1.2.24 Merdisoma\_sp934402125

Identified as 1 potentially new species

##### 1.2.24.0.1 16 phylogeny

##### 1.2.24.0.2 dDDH

### 1.2.25 Oribacterium\_sp002449915

Identified as 1 potentially new species

##### 1.2.25.0.1 16 phylogeny

##### 1.2.25.0.2 dDDH

### 1.2.26 UBA2821\_sp902763335

Identified as 1 potentially new species

##### 1.2.26.0.1 16 phylogeny

##### 1.2.26.0.2 dDDH

### 1.2.27 new species 36

Identified as 1 potentially new species

##### 1.2.27.0.1 16 phylogeny

##### 1.2.27.0.2 dDDH

### 1.2.28 new species 37

Identified as 1 potentially new species

##### 1.2.28.0.1 16 phylogeny

##### 1.2.28.0.2 dDDH

### 1.2.29 new species 38

Identified as 1 potentially new species

##### 1.2.29.0.1 16 phylogeny

##### 1.2.29.0.2 dDDH

### 1.2.30 new species 39

Identified as 1 potentially new species

##### 1.2.30.0.1 16 phylogeny

##### 1.2.30.0.2 dDDH

### 1.2.31 new species 40

Identified as 1 potentially new species

##### 1.2.31.0.1 16 phylogeny

##### 1.2.31.0.2 dDDH
