# Supplementary material for: Copper is an intestinal habitat filter affecting the gut microbiota interactions with Salmonella Typhimurium
Source: Microbiome. 2026 Mar 28;14:110. doi: 10.1186/s40168-025-02322-4 (PMC13064011; doi:10.1186/s40168-025-02322-4)
Supplement: Supplementary file 3 — Supplementary Material 2. Dataset S2 [file 40168_2025_2322_MOESM2_ESM.html]

 

 

 

 
 
 


 

 

 Strain diversity in cultured pig microbiota 

 
 
 
 
 
 
 
 
 
 
 
 
 
 
 
 
 
 
 
 
 
 
 
 
 
 
 
 
 
 
 
 
 
 
 
 
 

 

 
 


 


 

 

 


 


 

 


 


 
 
 
 
 
 

 


 


 Strain diversity in cultured pig
microbiota 
 Rafal Kolenda 
 2025-03-30 

 


 
 
  1  List of species with
more than 1 isolate: 
 
  
 
 
 
 
  2  Phylogeny, pangenomes
and SNP distances 
 
  2.1   Acidaminococcus
fermentans  
 
  2.1.1  SNP distance 
   
 
 
  2.1.2  Phylogeny and
pangenomes 
   
 
 
 
  2.2   Acidaminococcus
sp000437815  
 
  2.2.1  SNP distance 
   
 
 
 
 
 
 
 
  2.3   Agathobacter
faecis  
 
  2.3.1  SNP distance 
   
 
 
  2.3.2  Phylogeny and
pangenomes 
   
 
 
 
  2.4 
 Anaerobiospirillum succiniciproducens  
 
  2.4.1  SNP distance 
   
 
 
  2.4.2  Phylogeny and
pangenomes 
   
 
 
 
  2.5   Anaerofustis
stercorihominis  
 
  2.5.1  SNP distance 
   
 
 
 
 
 
 
 
  2.6   Bacillus
licheniformis  
 
  2.6.1  SNP distance 
   
 
 
 
 
 
 
 
  2.7   Bariatricus
sp004560705  
 
  2.7.1  SNP distance 
   
 
 
  2.7.2  Phylogeny and
pangenomes 
   
 
 
 
  2.8   Bifidobacterium
animalis  
 
  2.8.1  SNP distance 
   
 
 
  2.8.2  Phylogeny and
pangenomes 
   
 
 
 
  2.9   Bifidobacterium
boum  
 
  2.9.1  SNP distance 
   
 
 
  2.9.2  Phylogeny and
pangenomes 
   
 
 
 
  2.10   Bifidobacterium
catenulatum  
 
  2.10.1  SNP distance 
   
 
 
  2.10.2  Phylogeny and
pangenomes 
   
 
 
 
  2.11   Bifidobacterium
pseudolongum  
 
  2.11.1  SNP distance 
   
 
 
 
 
 
 
 
  2.12   Bifidobacterium
thermophilum  
 
  2.12.1  SNP distance 
   
 
 
  2.12.2  Phylogeny and
pangenomes 
   
 
 
 
  2.13   Bilifractor
sp002394235  
 
  2.13.1  SNP distance 
   
 
 
  2.13.2  Phylogeny and
pangenomes 
   
 
 
 
  2.14   Blautia_A 
unknown species 
 
  2.14.1  SNP distance 
   
 
 
  2.14.2  Phylogeny and
pangenomes 
   
 
 
 
  2.15   Blautia_A
massiliensis  
 
  2.15.1  SNP distance 
   
 
 
 
 
 
 
 
  2.16   Blautia_A
sp000285855  
 
  2.16.1  SNP distance 
   
 
 
  2.16.2  Phylogeny and
pangenomes 
   
 
 
 
  2.17   Blautia_A
sp003471165  
 
  2.17.1  SNP distance 
   
 
 
 
 
 
 
 
  2.18   Bulleidia
sp900539965  
 
  2.18.1  SNP distance 
   
 
 
  2.18.2  Phylogeny and
pangenomes 
   
 
 
 
  2.19   CAG-791
sp000431495  
 
  2.19.1  SNP distance 
   
 
 
  2.19.2  Phylogeny and
pangenomes 
   
 
 
 
  2.20   CAG-964
sp902789345  
 
  2.20.1  SNP distance 
   
 
 
  2.20.2  Phylogeny and
pangenomes 
   
 
 
 
  2.21   CAKVON01
sp934792625  
 
  2.21.1  SNP distance 
   
 
 
 
 
 
 
 
  2.22   Catenibacterium
mitsuokai  
 
  2.22.1  SNP distance 
   
 
 
  2.22.2  Phylogeny and
pangenomes 
   
 
 
 
  2.23   Citrobacter
braakii  
 
  2.23.1  SNP distance 
   
 
 
 
 
 
 
 
  2.24   Clostridium_F
sp001276215  
 
  2.24.1  SNP distance 
   
 
 
 
 
 
 
 
  2.25   Clostridium
sp012519155  
 
  2.25.1  SNP distance 
   
 
 
  2.25.2  Phylogeny and
pangenomes 
   
 
 
 
  2.26   Clostridium_F
sporogenes  
 
  2.26.1  SNP distance 
   
 
 
  2.26.2  Phylogeny and
pangenomes 
   
 
 
 
  2.27   Collinsella
bouchesdurhonensis  
 
  2.27.1  SNP distance 
   
 
 
  2.27.2  Phylogeny and
pangenomes 
   
 
 
 
  2.28   Collinsella
sp002391315  
 
  2.28.1  SNP distance 
   
 
 
  2.28.2  Phylogeny and
pangenomes 
   
 
 
 
  2.29   Dorea_A
longicatena  
 
  2.29.1  SNP distance 
   
 
 
 
 
 
 
 
  2.30   Enterococcus_E
cecorum  
 
  2.30.1  SNP distance 
   
 
 
 
 
 
 
 
  2.31 
 Escherichia  
 
  2.31.1  SNP distance 
   
 
 
  2.31.2  Phylogeny and
pangenomes 
   
 
 
 
  2.32   Eubacterium_T
pyruvativorans  
 
  2.32.1  SNP distance 
   
 
 
 
 
 
 
 
  2.33   Faecalicoccus
sp900546545  
 
  2.33.1  SNP distance 
   
 
 
  2.33.2  Phylogeny and
pangenomes 
   
 
 
 
  2.34   Fundicoccus 
unknown species 
 
  2.34.1  SNP distance 
   
 
 
 
 
 
 
 
  2.35   Fusicatenibacter
saccharivorans  
 
  2.35.1  SNP distance 
   
 
 
  2.35.2  Phylogeny and
pangenomes 
   
 
 
 
  2.36 
 Gracilibacillus  unknown species 
 
  2.36.1  SNP distance 
   
 
 
 
 
 
 
 
  2.37   Holdemanella
porci  
 
  2.37.1  SNP distance 
   
 
 
  2.37.2  Phylogeny and
pangenomes 
   
 
 
 
  2.38   Hornefia
butyriciproducens  
 
  2.38.1  SNP distance 
   
 
 
  2.38.2  Phylogeny and
pangenomes 
   
 
 
 
  2.39   Jeotgalibaca
porci  
 
  2.39.1  SNP distance 
   
 
 
 
 
 
 
 
  2.40   Lactobacillus
amylovorus  
 
  2.40.1  SNP distance 
   
 
 
  2.40.2  Phylogeny and
pangenomes 
   
 
 
 
  2.41   Lawsonibacter
sp944385035  
 
  2.41.1  SNP distance 
   
 
 
  2.41.2  Phylogeny and
pangenomes 
   
 
 
 
  2.42 
 Ligilactobacillus ruminis  
 
  2.42.1  SNP distance 
   
 
 
  2.42.2  Phylogeny and
pangenomes 
   
 
 
 
  2.43 
 Ligilactobacillus salivarius  
 
  2.43.1  SNP distance 
   
 
 
  2.43.2  Phylogeny and
pangenomes 
   
 
 
 
  2.44 
 Limosilactobacillus reuteri  
 
  2.44.1  SNP distance 
   
 
 
 
 
 
 
 
  2.45 
 Mediterraneibacter faecis  
 
  2.45.1  SNP distance 
   
 
 
  2.45.2  Phylogeny and
pangenomes 
   
 
 
 
  2.46   Merdisoma
sp934402125  
 
  2.46.1  SNP distance 
   
 
 
 
 
 
 
 
  2.47   Niallia
sp001076885  
 
  2.47.1  SNP distance 
   
 
 
 
 
 
 
 
  2.48   Oliverpabstia
intestinalis  
 
  2.48.1  SNP distance 
   
 
 
  2.48.2  Phylogeny and
pangenomes 
   
 
 
 
  2.49   Oribacterium
sp004554245  
 
  2.49.1  SNP distance 
   
 
 
 
 
 
 
 
  2.50   Paenibacillus
A_barengoltzii  
 All three isolates are identical 
 
 
  2.51   Parafannyhessea
sp900538935  
 
  2.51.1  SNP distance 
   
 
 
 
 
 
 
 
  2.52   Paralachnospira
sp934351765  
 
  2.52.1  SNP distance 
   
 
 
 
 
 
 
 
  2.53   Phocaeicola
vulgatus  
 
  2.53.1  SNP distance 
   
 
 
  2.53.2  Phylogeny and
pangenomes 
   
 
 
 
  2.54   Prevotella
copri_A  
 
  2.54.1  SNP distance 
   
 
 
  2.54.2  Phylogeny and
pangenomes 
   
 
 
 
  2.55   Prevotella
faecis  
 
  2.55.1  SNP distance 
   
 
 
 
 
 
 
 
  2.56   Prevotella
sp900546535  
 
  2.56.1  SNP distance 
   
 
 
 
 
 
 
 
  2.57   Prevotella
sp945863825  
 
  2.57.1  SNP distance 
   
 
 
 
 
 
 
 
  2.58   RUG115
sp900315735  
 
  2.58.1  SNP distance 
   
 
 
 
 
 
 
 
  2.59   Sarcina
perfringens  
 
  2.59.1  SNP distance 
   
 
 
  2.59.2  Phylogeny and
pangenomes 
   
 
 
 
  2.60   Selenomonas_A
montiformis  
 
  2.60.1  SNP distance 
   
 
 
 
 
 
 
 
  2.61   Streptococcus
alactolyticus  
 
  2.61.1  SNP distance 
   
 
 
  2.61.2  Phylogeny and
pangenomes 
   
 
 
 
  2.62   UBA1417
sp003531055  
 
  2.62.1  SNP distance 
   
 
 
  2.62.2  Phylogeny and
pangenomes 
   
 
 
 
  2.63   UBA2821
sp902763335  
 
  2.63.1  SNP distance 
   
 
 
  2.63.2  Phylogeny and
pangenomes 
   
 
 
 
  2.64   UBA7741
sp900314575  
 
  2.64.1  SNP distance 
   
 
 
  2.64.2  Phylogeny and
pangenomes 
   
 
 
 
  2.65   VUNA01
sp002299625  
 
  2.65.1  SNP distance 
   
 
 
 


 
 

 

 

 

 

 

 

 
 

 
 
