# Supplementary material for: Copper is an intestinal habitat filter affecting the gut microbiota interactions with Salmonella Typhimurium
Source: Microbiome. 2026 Mar 28;14:110. doi: 10.1186/s40168-025-02322-4 (PMC13064011; doi:10.1186/s40168-025-02322-4)
Supplement: Supplementary file 4 — Supplementary Material 3. Figure S1-S11 [file 40168_2025_2322_MOESM3_ESM.docx]

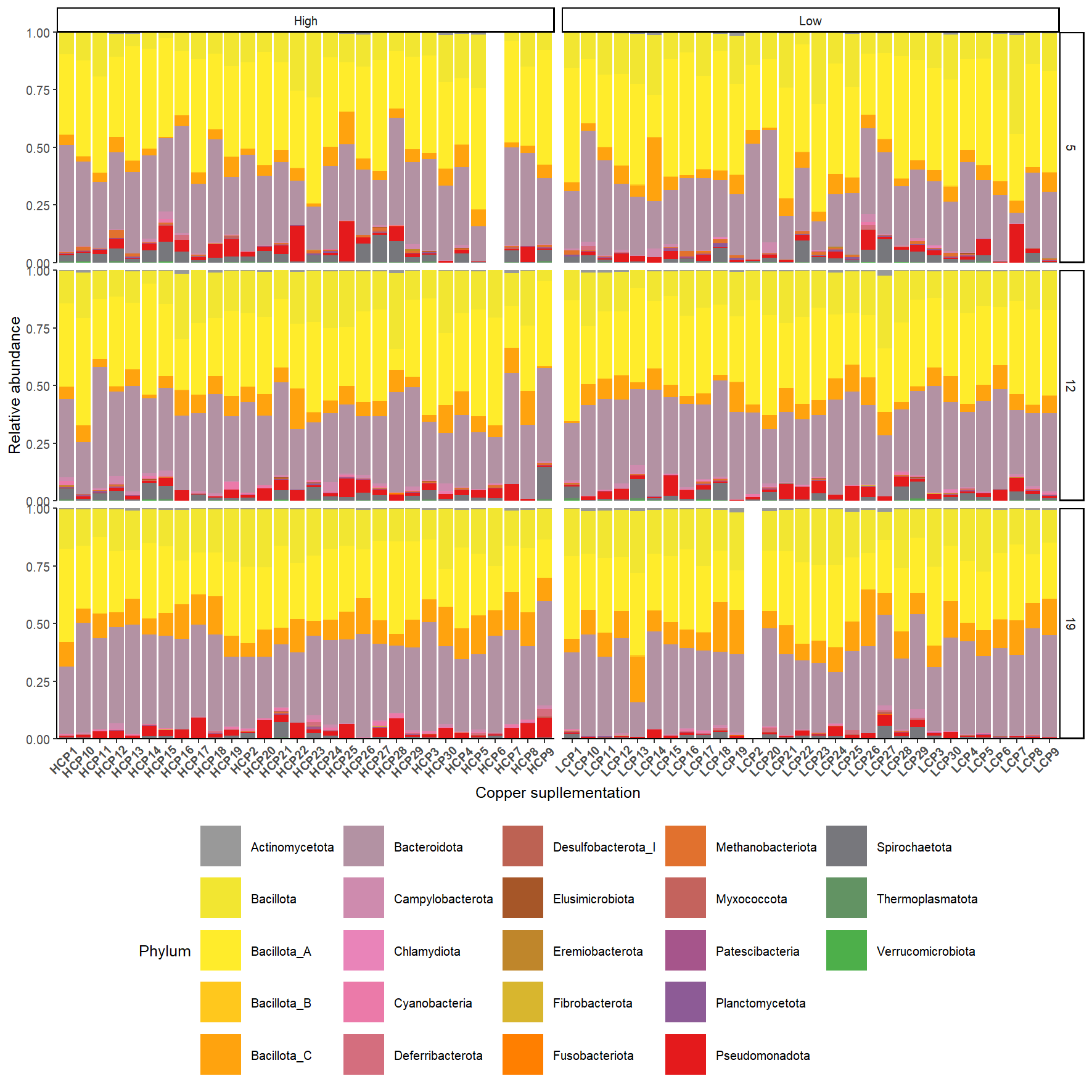


Figure S1. Relative abundance of phyla in piglets

Stacked barplot with relative abundance of phyla in all faecal samples for piglets from the farm study. Animal groups are shown on the top x-axis and animals on the bottom x-axis. Animal study day is marked on the right y-axis and relative abundance is shown on the left y-axis. Colours correspond to the specific phyla shown on the legend.


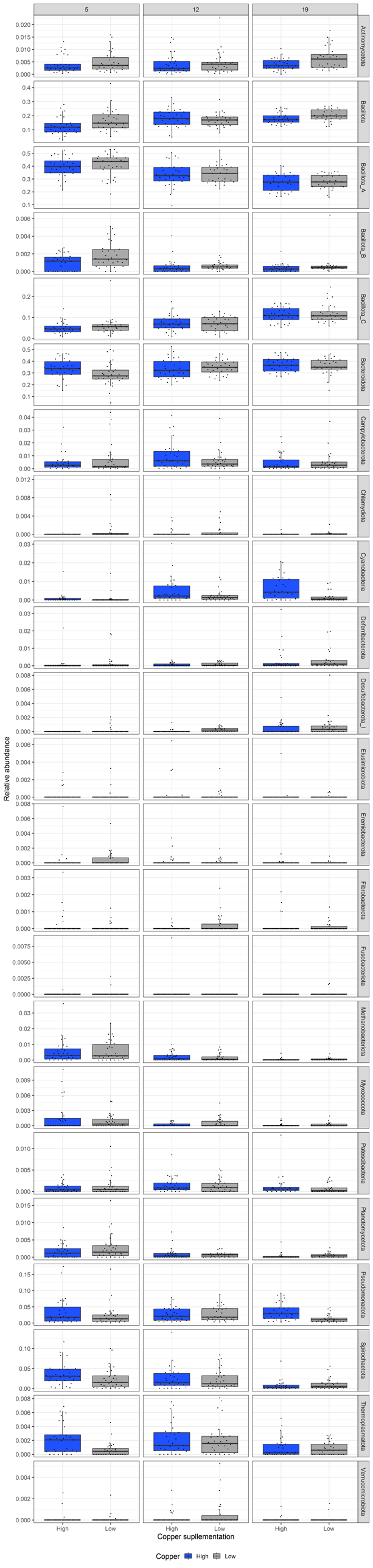


Figure S2. Relative abundance of phyla in piglets

Whisker Box-dotplot with relative abundance of phyla in all faecal samples for piglets from the farm study. Animal groups are shown on the bottom x-axis and coloured and shown on the legend and Animal study day on the top x-axis. Phylum named is marked on the right y-axis and relative abundance is shown on the left y-axis. Each dot represents relative abundance for one piglet from one group at one timepoint.


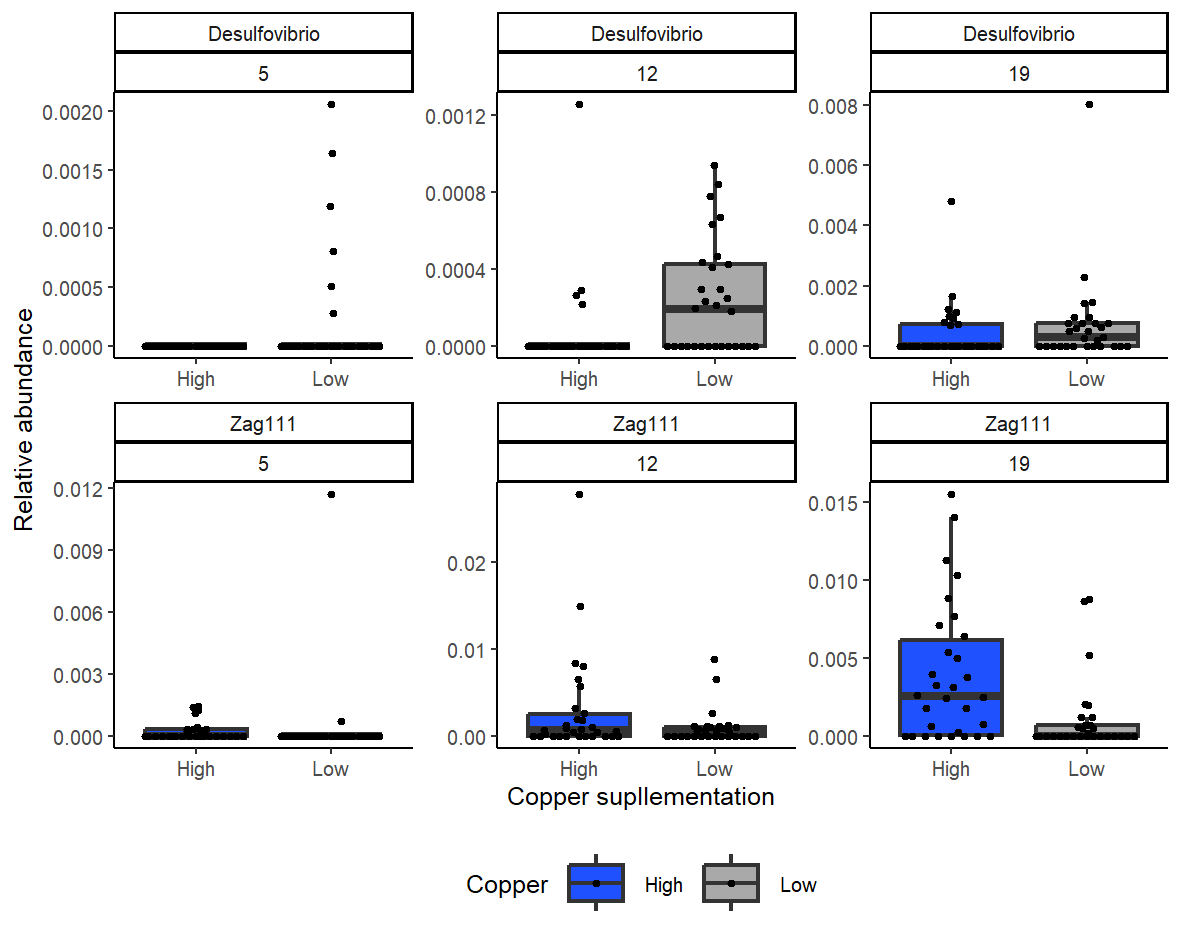


Figure S3. Relative abundance of selected genera in piglets

Whisker Box-dotplot with relative abundance of genus in all faecal samples for piglets from the farm study. Animal groups are shown on the bottom x-axis and coloured and shown on the legend and Animal study day and genus on the top x-axis. Relative abundance is shown on the left y-axis. Each dot represents relative abundance for one piglet from one group at one timepoint.


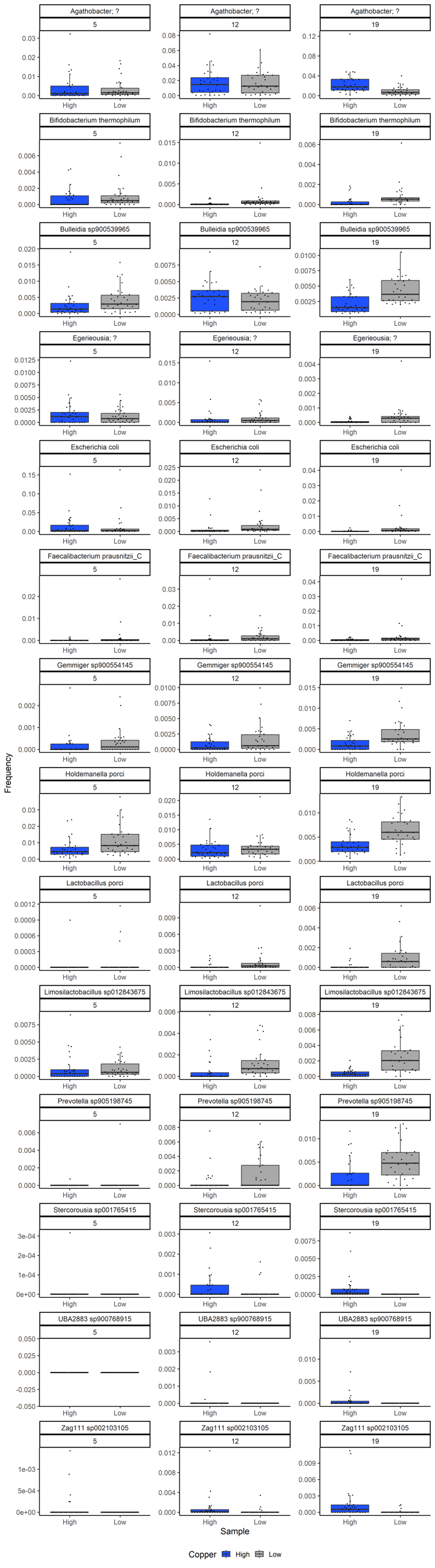


Figure S4. Relative abundance of species statistically significant differences between piglets on low and high copper diet

Whisker Box-dotplot with relative abundance of species in all faecal samples for piglets from the farm study. Animal groups are shown on the bottom x-axis and coloured and shown on the legend and Animal study day and species on the top x-axis. Relative abundance is shown on the left y-axis. Each dot represents relative abundance for one piglet from one group at one timepoint.


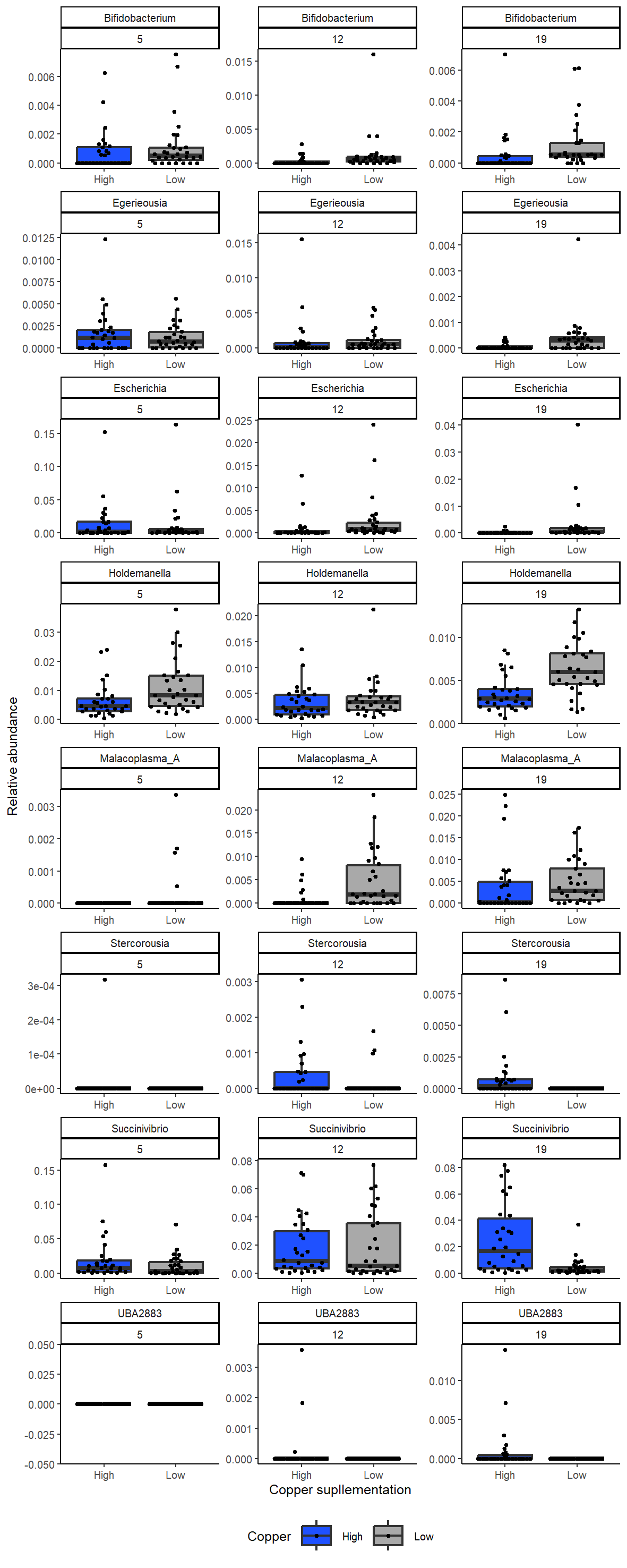


Figure S5. Relative abundance of genera statistically significant differences between piglets on low and high copper diet

Whisker Box-dotplot with relative abundance of genera in all faecal samples for piglets from the farm study. Animal groups are shown on the bottom x-axis and coloured and shown on the legend and Animal study day and genus on the top x-axis. Relative abundance is shown on the left y-axis. Each dot represents relative abundance for one piglet from one group at one timepoint.


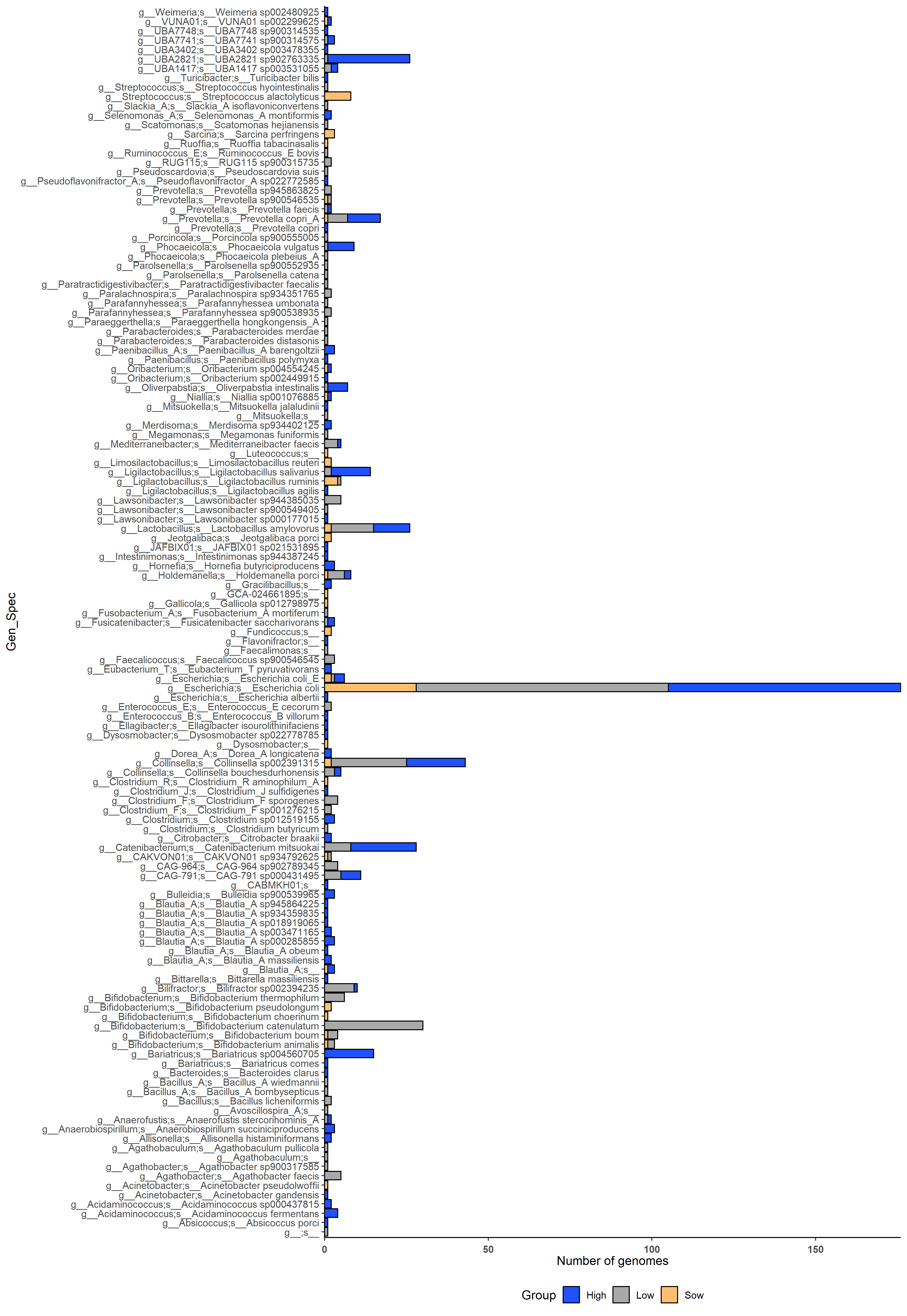


Figure S6. Number of isolates from cultured microbiota

Stacked barplot with isolate count for species isolated from 6 piglets and 1 sow. Colours correspond to animal groups and are shown on the legend. Isolate counts are shown on the x-axis. Species name is shown on the left y-axis.


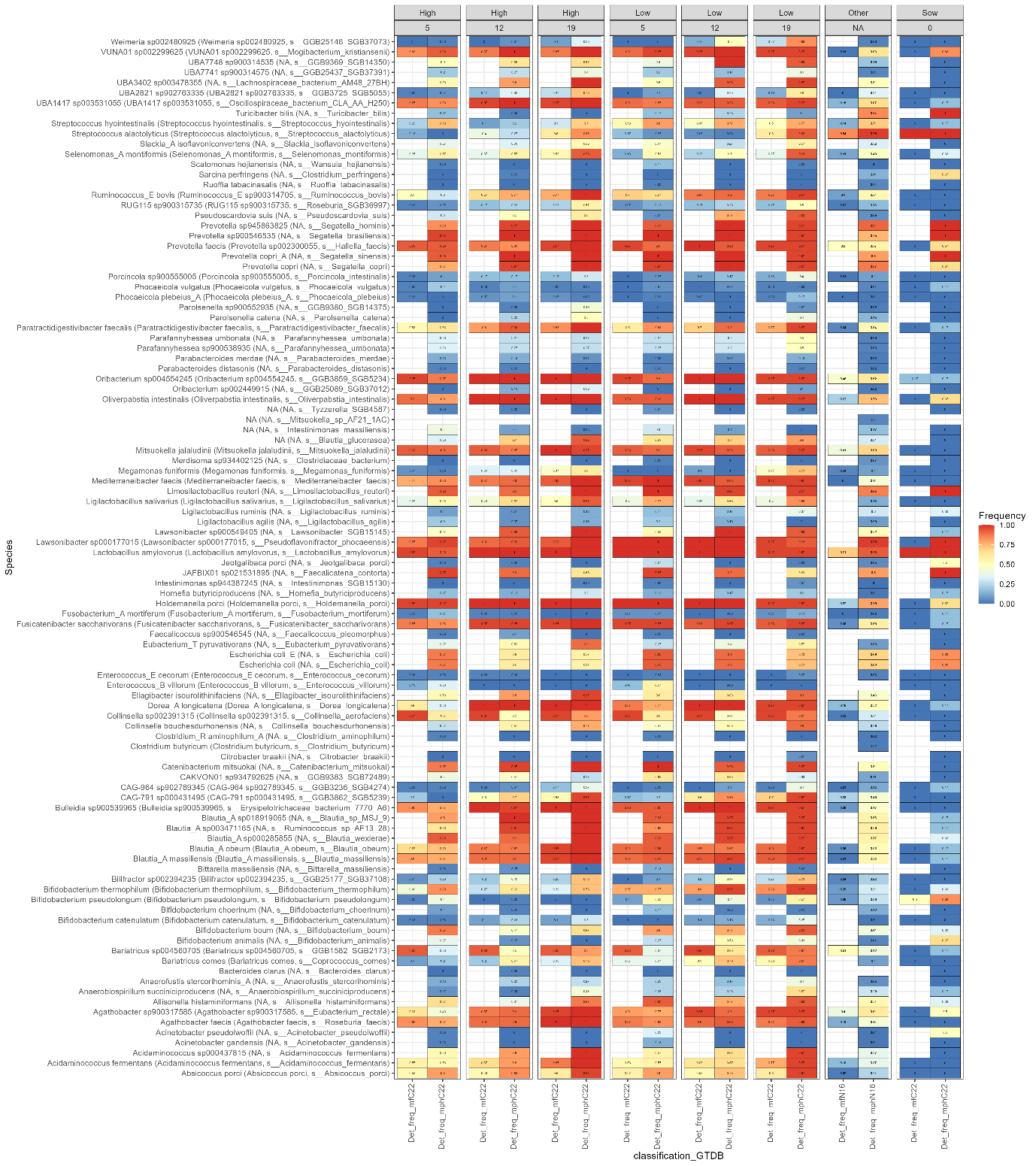


Figure S7. Frequency of detection of cultured microbiota in pig metagenomics data

Heatmap with species detection frequences in pig metagenomics data. Colours correspond to frequency of species detection (0- no detection, 1- species detected in all samples) and are shown on the legend. Datasets and detection methods are shown on the bottom x-axis (mf- MATAFILER, mph- Metaphlan4.1.1, C22- this study, N16- PRJEB11755 (Xiao et al.,2016) ). Animal study day and group/external data(marked as “Other”) are shown on the top x-axis. Species names is shown on the y-axis [formatting is as follows GTDB-tk_v214(GTDB-tk_v207, metaphlan_v4.1.1_ database mpa_vJun23_CHOCOPhlAnSGB_202403)].


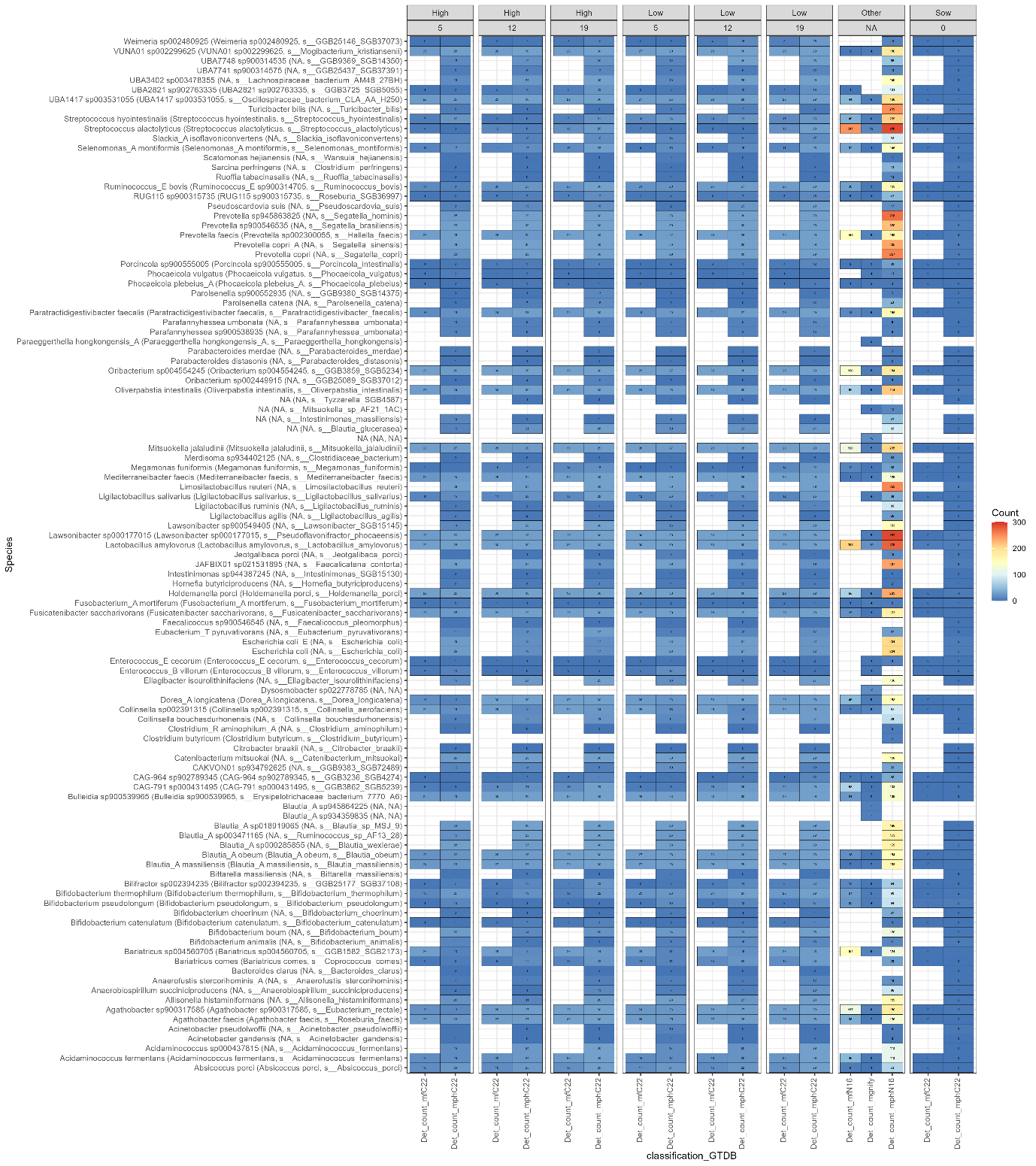


Figure S8. Detection of cultured microbiota in pig metagenomics data

Heatmap with species detection counts in pig metagenomics data. Colours correspond to no. of positive samples with a species and are shown on the legend. Datasets and detection methods are shown on the bottom x-axis (mf- MATAFILER, mph- Metaphlan4.1.1, C22- this study, N16- PRJEB11755 (Xiao et al.,2016), mgnify - Pig Gut v1.0 MGnify Genome database). Animal study day and group/external data(marked as “Other”) are shown on the top x-axis. Species names is shown on the y-axis [formatting is as follows GTDB-tk_v214(GTDB-tk_v207, metaphlan_v4.1.1_ database mpa_vJun23_CHOCOPhlAnSGB_202403)].


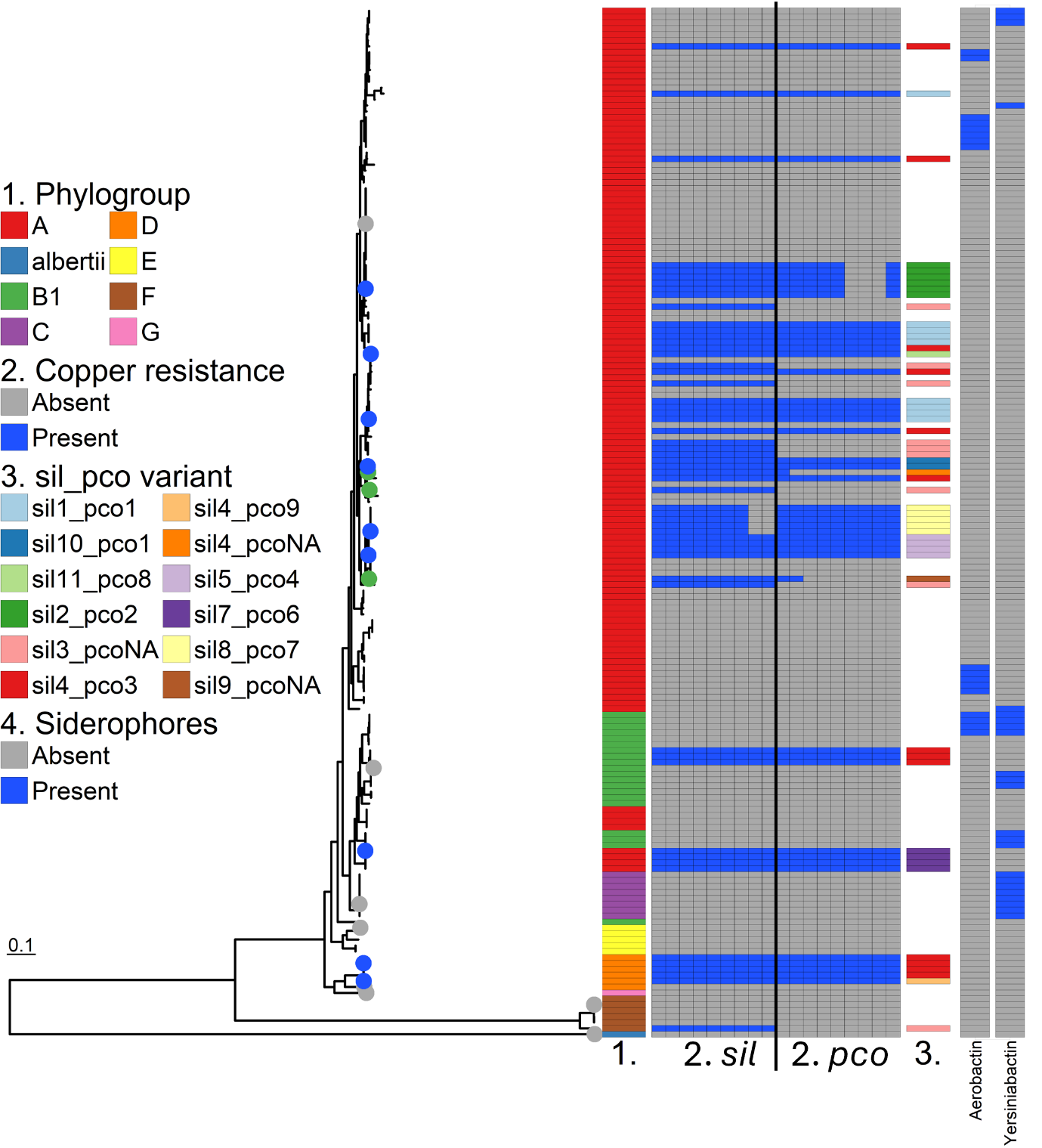


Figure S9. Presence of selected siderophores in pig *E. coli*

Phylogenetic tree based on core genome SNPs of 174 *Escherichia* isolates in this study annotated with phylogroup, *sil*/*pco* genes and *sil*/*pco* gene cluster variants and presence of two sireophores – Aerobactin and Yersiniabactin. Strains selected for copper sulphate broth microdilution assays are marked with blue (*sil/pco*-positive, n=9), green (*sil*-positive, n=3) or grey dot (*sil/pco*-negative, n=8).


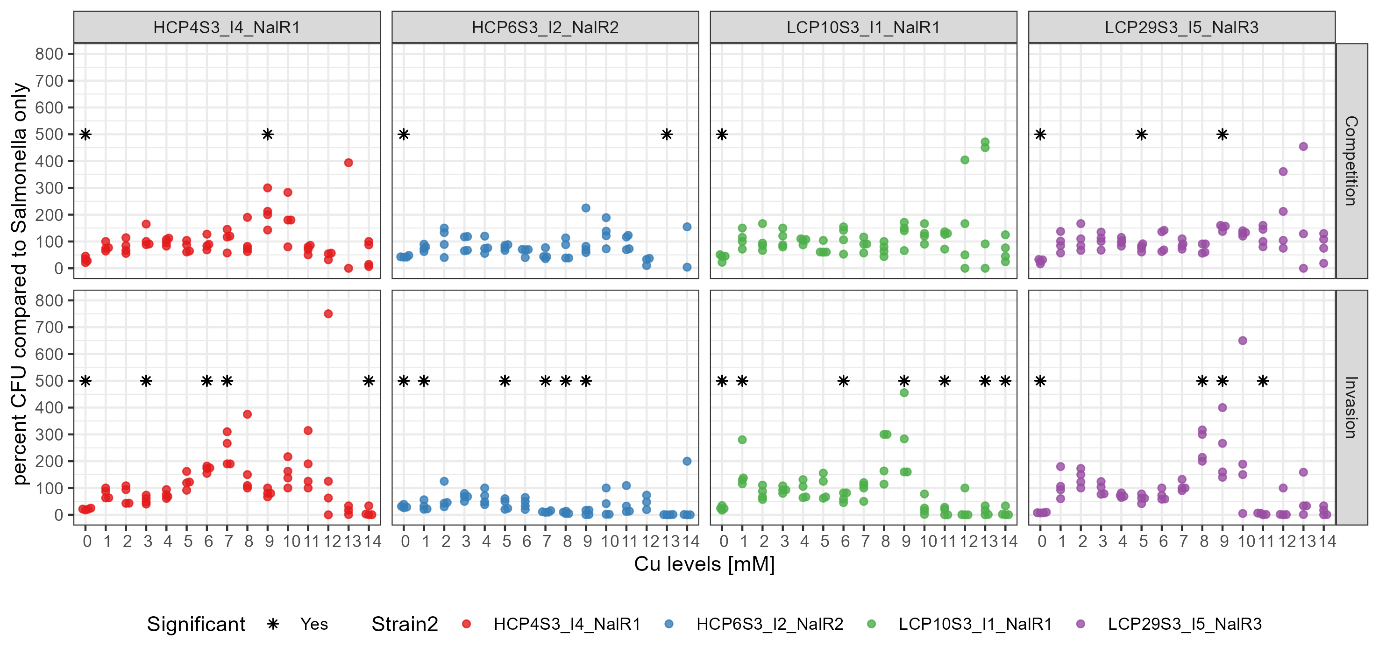


Figure S10. Effect of *Escherichia* isolates on *Salmonella* growth

Dotplots showing relative CFUs of *Salmonella* after 24 h incubation with various *Escherichia* isolates in anaerobic conditions in comparison with CFU of *Salmonella*  monocultures. Significant difference between CFU of *Salmonella* in co-culture and monoculture are marked with asterix (Wilcoxon, p < 0.05).


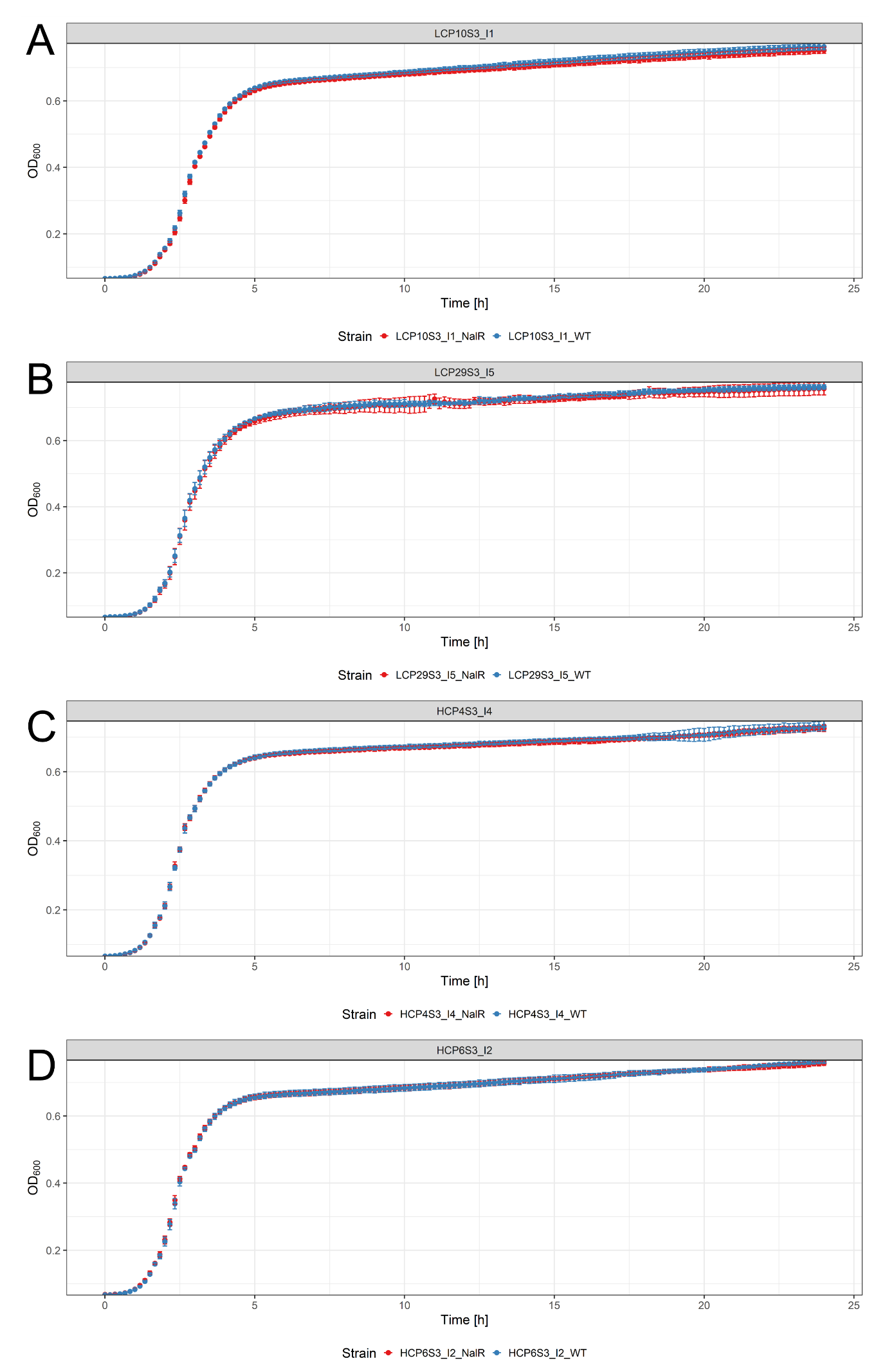


Fig. S11. Effect of spontaneous nalidixic acid mutation on growth of *E. coli* strains.

Strain A) LCP10S3_I1 wild type (LCP10S3_I1_WT) and its derivate with spontaneous nalidixic acid mutation (LCP10S3_I1_NalR), B) LCP29S3_I5 wild type (LCP29S3_I5_WT) and its derivate with spontaneous nalidixic acid mutation (LCP29S3_I5_NalR), C) HCP4S3_I4 wild type (HCP4S3_I4_WT) and its derivate with spontaneous nalidixic acid mutation (HCP4S3_I4_NalR), D) HCP6S3_I2 wild type (HCP6S3_I2_WT) and its derivate with spontaneous nalidixic acid mutation (HCP6S3_I2_NalR) growth curve in LB for 24 h. Time [hours] is shown on the x-axis and optical density at 600 nm (OD_600_) is shown on the y-axis. Each dot and error bar corresponds to one timepoint. Dots and errorbars colour correspond to isolates and are shown on the legend below each figure.
